# Let’s Escape Didactics: Virtual Escape Room as a Didactic Modality in Residency

**DOI:** 10.21980/J8CH2X

**Published:** 2021-04-19

**Authors:** Anisha Turner, Aleksandr M Tichter, M Tyson Pillow

**Affiliations:** * Baylor College of Medicine, Department of Emergency Medicine, Houston, Tx

## Abstract

**Audience:**

The virtual escape room is a didactic activity for emergency medicine residents (interns, junior residents, senior residents).

**Introduction:**

Residency programs are employing a wide variety of active learning techniques to engage their learners, including large-group discussion, small-group activities, team-based learning, gamification, problem-based learning, role-playing and case studies. In recent years, educators have drawn their attention to educational escape rooms, a new type of learning activity that utilizes collaborative learning activities to foster creating thinking, communication, teamwork and leadership.^1^–^3^ There have been a number of cases in medicine, 4,5 but there have been limited works published on the use of virtual educational escape rooms in residency education.

Unfortunately, the COVID pandemic has made participation in an escape room more difficult. In lieu of social distancing during the COVID pandemic, participation in a virtual escape room is an effective and flexible learning modality for resident didactics that appears to promote participant satisfaction, competency, learning, and engagement.

**Educational Objectives:**

By the end of the activity, learners should be able to:

1. Identify the hazardous chemicals associated with house fires
2. Classify burn injury according to depth, extent and severity based on established standards
3. Recall the actions to take in response to fire emergencies (R.A.C.E. and P.A.S.S. acronyms)
4. Recall key laboratory features of cyanide and carbon monoxide poisonings
5. Identify appropriate management strategies for smoke inhalation injuries
6. Recite the treatment for cyanide and carbon monoxide poisonings
7. Describe the management of the burn injuries
8. Communicate and collaborate as a team to arrive at solutions of problems
9. Display task-switching and leadership skills during exercise
10. Evaluate virtual escape room experience

**Educational Methods:**

Emergent care of burns, a popular and shared topic in both Emergency Medicine and Family Medicine literature, was chosen and educational objectives were developed. The website Deck.Toys was utilized to formulate the escape room along with puzzles around the educational objectives. Students congregated remotely on Zoom, and after instructions, were separated into teams to solve content-specific puzzles in order to escape the room. Teams which solve all the puzzles in the allotted time were considered to have successfully escaped the room. After the allotted time, the faculty led debriefing, and topic discussion occurred.

**Research Methods:**

Sixty-three participants composed of residents (24 emergency [EM], 29 family medicine [FM], 4 combined emergency and family medicine [EM/FM]), advanced practice practitioner trainees (2 EM), and faculty member participants (4 FMP) partook in the virtual escape room experience. At the end of the activity, a 17-item survey using Likert-scale questions was embedded in order to obtain feedback regarding satisfaction, engagement, learning, and medical competency in communication, collaboration, task-switching, and leadership skills.

**Results:**

Eighteen out of 63 participants filled out the survey. This was the first virtual escape room experience for 94% of the respondents. A majority (88.9%) of respondents enjoyed the virtual escape room, finding it fun, interesting, engaging, and interactive. None of the respondents preferred traditional didactics over the virtual escape room activity, and 72% were either just as or equally as satisfied with virtual compared to in-person escape rooms. Nearly all respondents agreed that the activity encouraged collaboration, communication skills, task-switching, and leadership skills (94.4%, 88.9%, 72.2%, 72.2%, respectively).

**Discussion:**

Participation in a virtual escape room is an effective and flexible learning modality for resident didactics that appears to promote learner satisfaction and engagement. The escape room also promoted important competencies encouraged during residency, such as interpersonal and communication skills and practice-based learning and improvement, and is an effective addition to virtual learning tools.

**Topics:**

Small group activity, team-building exercise, remote learning, virtual learning, educational games, gamification, medical education, escape room, millennials, student engagement, adult learning theory, emergency medicine residents, family medicine residents, chemicals in house fires, smoke inhalation injuries, burn classification, burn injury management, carbon monoxide poisoning, cyanide poisoning, R.A.C.E. acronym, P.A.S.S. acronym, fluid resuscitation in burn patients, burn referrals.

## USER GUIDE

[Table t2-jetem-6-2-sg46]

**Table t2-jetem-6-2-sg46:** 

**List of Resources:**	
Abstract	46
User Guide	48
Appendix A: The Virtual Escape Room Burn and Inhalation Injuries	56

**Learner Audience:**

Interns, Junior Residents, Senior Residents, Advanced Practice Provider Trainees, Faculty

While the level of learners was not documented, there were sixty-three participants in total. There was a total of 57 residents (24 EM, 29 FM, 4 EMFM), 2 advanced practice practitioner trainees (EM), and 2 faculty member participants (FMP).

**Time Required for Implementation:**

The Deck.Toys platform will take approximately 1–5 hours prior to the session based on user experience and familiarity with the platform as well as ability to formulate puzzles and develop content. Time can be saved by accessing the premade Deck Toy template utilized in this exercise and making changes to the activities as desired (website: https://deck.toys/a/XkDiDc2IK). The actual didactic session can be tailored to 60 to 75-minute sessions depending on the learner’s technical ability and familiarity with the content. Ten minutes should be allotted to activity introduction, 15–30 minutes for learners to complete the escape room depending on learner’s familiarity with content, 5 minutes for a short debrief session, and 30 minutes for topic presentation and discussion.

**Recommended Number of Learners per Instructor**:

One instructor should be able to oversee the entire exercise, from introduction to facilitation and discussion. Since this is a team-based and learner-led activity, the instructor can rotate through each small group to answer technical questions, ensure participants are following rules, and to observe team dynamics for feedback. To alleviate the instructor duties and be more effective, one volunteer (staff, students, faculty, fellows, chief residents) for each 4 to 6-member small group can be incorporated to oversee each virtual breakout room. These additional volunteers do not need background knowledge in emergency management of burn and smoke inhalation injuries. Volunteers were not utilized in our exercise, but are advised.

**Topics:**

Small group activity, team-building exercise, remote learning, virtual learning, educational games, gamification, medical education, escape room, millennials, student engagement, adult learning theory, emergency medicine residents, family medicine residents, chemicals in house fires, smoke inhalation injuries, burn classification, burn injury management, carbon monoxide poisoning, cyanide poisoning, R.A.C.E. acronym, P.A.S.S. acronym, fluid resuscitation in burn patients, burn referrals.

**Objectives:**

By the end of the activity, learners should be able to:

1. Identify the hazardous chemicals associated with house fires
2. Classify burn injury according to depth, extent and severity based on established standards
3. Recall the actions to take in response to fire emergencies (R.A.C.E. and P.A.S.S. acronyms)
4. Recall key laboratory features of cyanide and carbon monoxide poisonings
5. Identify appropriate management strategies for smoke inhalation injuries
6. Recite the treatment for cyanide and carbon monoxide poisonings
7. Describe the management of the burn injuries
8. Communicate and collaborate as a team to arrive at solutions of problems
9. Display task-switching and leadership skills during exercise
10. Evaluate virtual escape room experience

### Linked objectives and methods

Objective eight (communicate and collaborate), nine (task-switching and leadership skills) and ten (evaluate virtual escape room experience) is evident throughout the activity. Team members must communicate and use each other's knowledge and resourcefulness to arrive at solutions to each challenge. Participants must utilize task-switching skills to pay attention to comments made by team members while looking up information, complete tasks and activities whole communicating new solutions or hints during the exercise, and actively communicate with team members while analyzing each puzzle. Participants must utilize leadership skills in order to effectively operate in a team. Team collaboration and communication was apparent during the activity (see Activity 1 below). Additionally, students are asked to evaluate their experience at the end of the activity. Each of the remaining activities (1–7) are achieved in activities in the virtual escape room.[Table t3-jetem-6-2-sg46]

**Table t3-jetem-6-2-sg46:** 

Activity	Linked Objective
1. Crossword Puzzle	1, 8, 9
2. Matching	4, 5, 6, 8, 9
3. Sequence	3, 8, 9
4. Sequence	3, 8, 9
5. Multiple Choice	2, 6, 8, 9
6. Matching	2, 8, 9
7. Sorting	7, 8, 9
8. Short Answer	7, 8, 9

Knowledge is solidified during the answer and explanation session following the activity.

### Recommended pre-reading for facilitator

The instructor should have moderate background knowledge of emergency management of burn and smoke inhalation injuries (emergency physician, family medicine physician). The instructor should be familiar with Small Group Exercise Activity Answers & Pearls section of this document, which details the explanations/answers for each activity/challenge in the Deck.Toys platform. The information provided in the aforementioned should be sufficient. If more background information on the topic selected in this case is desired, the following would be helpful:

1. Scott Weingart. EMCrit RACC Podcast 219 – Critical Burn Patients in the ED/ICU – Part I with Dennis Djogovic. EMCrit Blog. Published on March 5, 2018. Accessed on May 21st 2019. Available at https://emcrit.org/emcrit/critical-burn-patients-in-the-ed/
2. Pham TN, Cancio LC, Gibran NS. American Burn Association Practice Guidelines: Burn Shock Resuscitation. *J of Burn Care and Research*. Jan–Feb 2008;29(1):257–266.
3. Jeschke MG, van Baar ME, Choudhry MA, Chung KK, Gibran NS, Logsetty S. Burn Injury. *Nat Rev Dis Primers*. 2020;6(1):11. Published 2020 Feb 13. doi:10.1038/s41572-020-0145-5
4. Koyfman A. The EM Educator Series: why is my burn patient so sick? emDocs. Published March 15, 2018. http://www.emdocs.net/wpcontent/uploads/2018/03/Educator-Download-Burn.pdf

### Associated Materials

- Activity PowerPoint:
  ○ Introduction Slides and Overview for discussion and wrap up
- DeckToy Activity
  ○ DeckToy platform in learner and teacher format
- Small Group Activity Exercise Answers & Pearls (below)
  ○ For facilitator to review with students if questions

### Learner responsible content (LRC)

Although not necessary, learners may benefit from the following resources if pre-preparation or pre-reading is desired:

1. Radwine Z. Carbon Monoxide Poisoning. *emDocs*. 2015, April, 26. http://www.emdocs.net/carbon-monoxide-poisoning/
2. Bridwell R, Cibrario A. EM@3AM: Cyanide Toxicity. *emDocs*. 2020, July, 17. http://www.emdocs.net/em3am-cyanide-toxicity/
3. Koyfman A. The EM Educator Series: Why is my burn patient so sick? e*mDocs*. 2018, Mar, 15. http://www.emdocs.net/wpcontent/uploads/2018/03/Educator-Download-Burn.pdf

**Small group application exercise:** Since there are multiple challenges in this didactic, they are explained in detail below:

### Equipment

Instructor Equipment

- Deck.Toys Membership (free)
- Electronic device (laptop, computer)
- Video Conferencing Application (Zoom)

Learner Equipment

- Participants were required to have an electronic device with internet capabilities.

### Instructor Instructions

#### Pre-Session

1. Join Deck.Toys as an instructor/teacher: https://deck.toys/
2. Access the instructor’s version and review: https://deck.toys/a/XkDiDc2IK
  - You may embed media gif images as desired
3. Review the PowerPoint and personalize slides as needed
4. Review reading materials

#### Session

1. Present PowerPoint’s introductory slides in 10 minutes (Slides 1–8)
2. Separate attendees into groups of 4–6 members, each group with an effort to preserve an equal distribution of learner experience based on level of training. Select a team leader for each group. (Optional: assign an observer to each group). Start Breakout Session for 15 minutes.
3. Return to large group and announce winner (Slide 9)
4. Debrief for 5 minutes (Slide 10). During this time, you can ask “How was the experience? What went well? What did not?”
5. Deliver topic content over 30 minutes. (Slides 11–35)
6. Deliver survey (Slide 36)

### Activities

#### Activity 1: Crossword Puzzle

[Fig f1-jetem-6-2-sg46]

**Figure f1-jetem-6-2-sg46:**
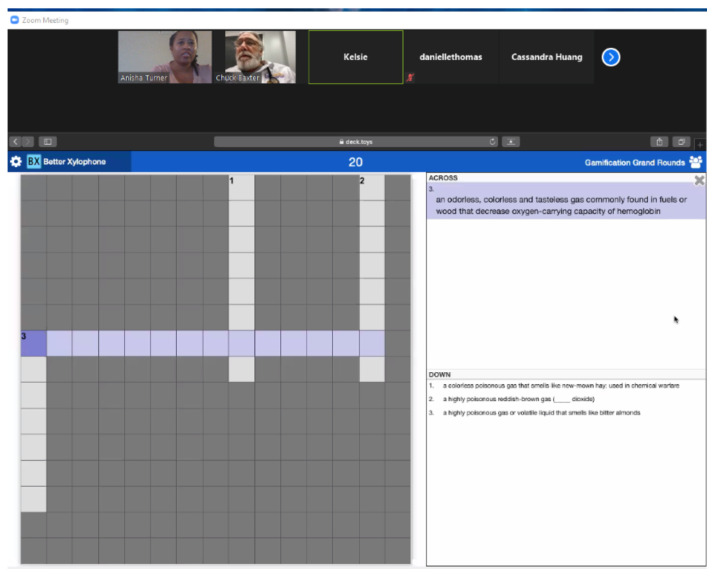


#### Activity 2: Matching

[Fig f2-jetem-6-2-sg46]

**Figure f2-jetem-6-2-sg46:**
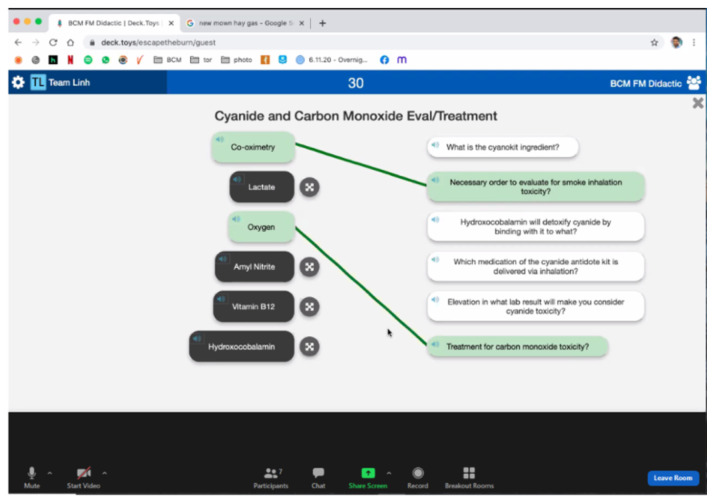


#### Activity 3: Sequence

[Fig f3-jetem-6-2-sg46]

**Figure f3-jetem-6-2-sg46:**
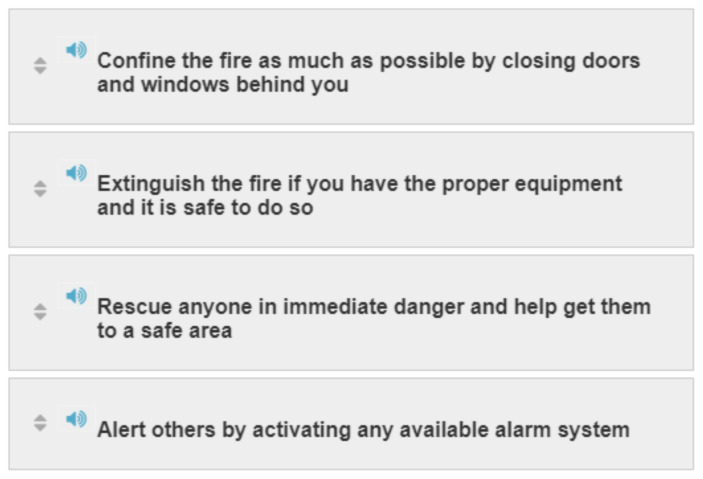


#### Activity 4: Sequence

[Fig f4-jetem-6-2-sg46]

**Figure f4-jetem-6-2-sg46:**
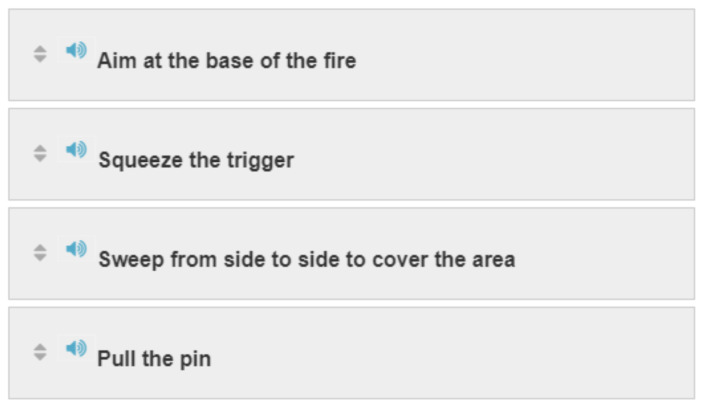


#### Activity 5: Multiple Choice

**Figure:**
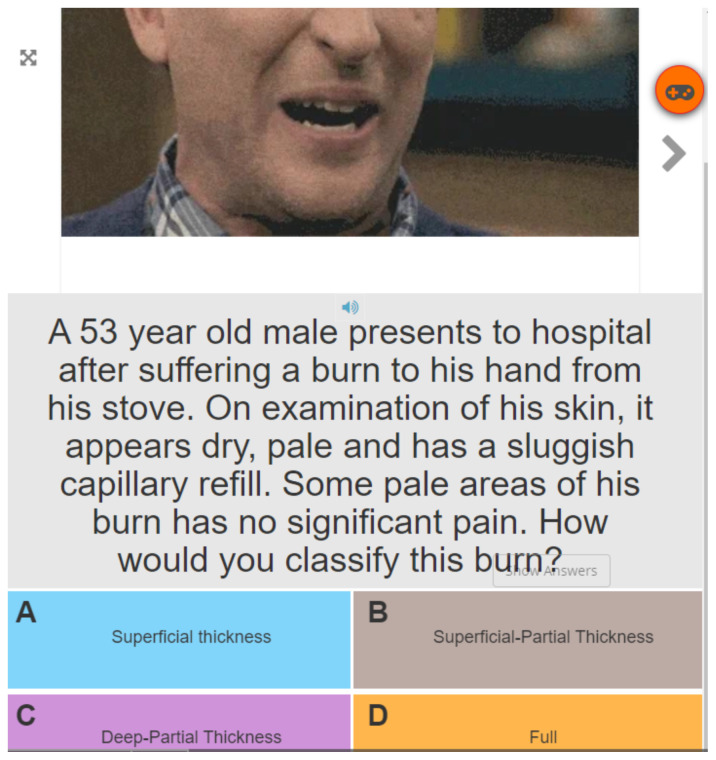


#### Activity 6: Matching

[Fig f6-jetem-6-2-sg46]

**Figure f6-jetem-6-2-sg46:**
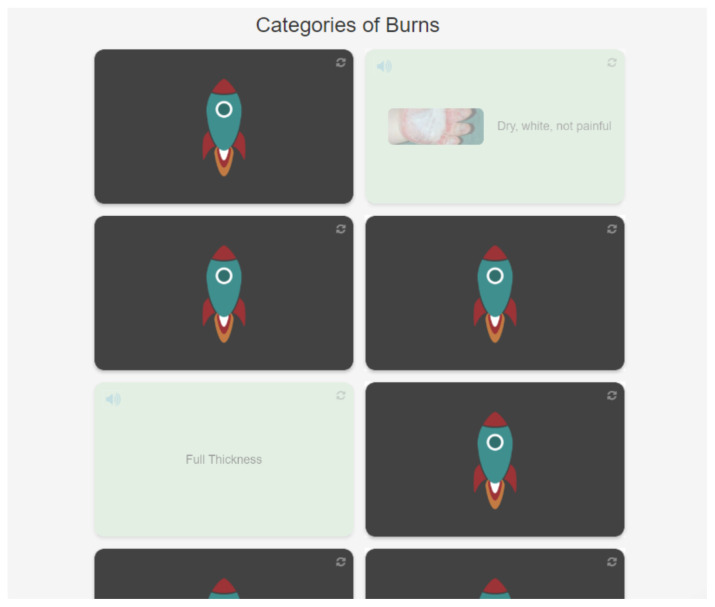


#### Activity 7: Sorting

[Fig f7-jetem-6-2-sg46]

**Figure f7-jetem-6-2-sg46:**
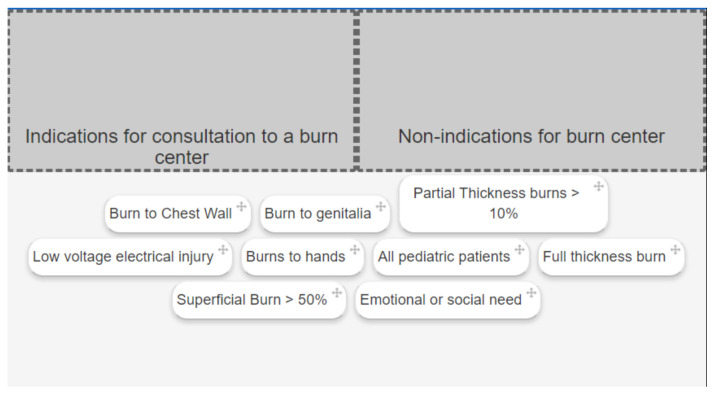


#### Activity 8: Short Answer

[Fig f8-jetem-6-2-sg46]

**Figure f8-jetem-6-2-sg46:**
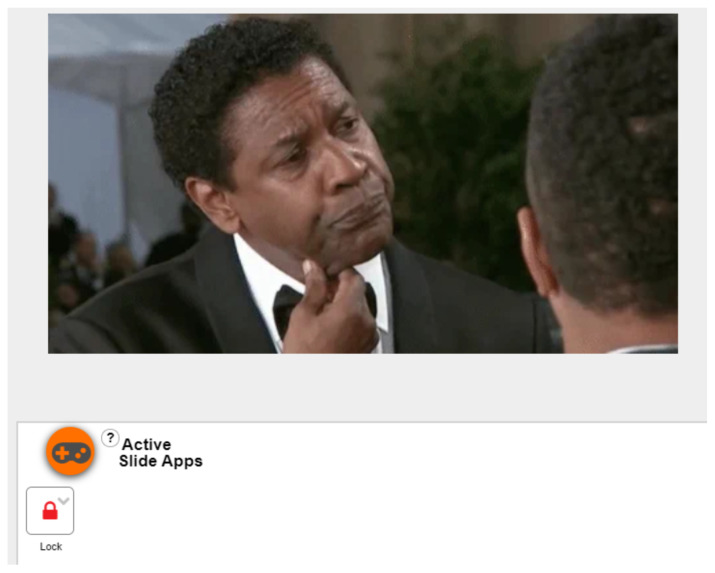


### Small Group Application Exercise Answers & Pearls

#### Activity 1

- Nitrogen Dioxide: a highly poisonous reddish-brown gas
- Cyanide: a highly poisonous gas or volatile liquid that smells like bitter almonds
- Carbon Monoxide: an odorless, colorless and tasteless gas commonly found in fuels or wood that decreases oxygen-carrying capacity of hemoglobin
- Phosgene: a colorless poisonous gas that smells like new-mown hay; used in chemical warfare

##### Pearl #1

Hazardous chemicals associated with house fires

- Nitrogen Dioxide: a highly poisonous reddish-brown gas, typically produced from the combustion of fabric. Formation of nitric oxide, a potent vasodilator, leads to increased bronchial blood flow, decreased hypoxic pulmonary vasoconstriction in poorly ventilated lung spaces, and finally hypoxia from V/Q mismatch.^16^
- Cyanide: a highly poisonous gas or volatile liquid that is developed from an incomplete combustion of any material containing nitrogen such as plastic, vinyl, wool, or silk. The gaseous form smells like bitter almonds.^12^
- Carbon Monoxide: an odorless, colorless and tasteless gas commonly found in fuels or wood that decreases oxygen-carrying capacity of hemoglobin and is the main toxic compound in fire deaths.^11^
- Phosgene: a colorless poisonous gas that smells like new-mown hay that is usually caused from burning of plastics or pesticides; used in chemical warfare.^16^

#### Activity 2

- Lactate: Elevation in what lab result will make you consider cyanide toxicity?
- Co-oximetry: Necessary order to evaluate for smoke inhalation toxicity?
- Amyl nitrite: Which medication of the cyanide antidote kit is delivered via inhalation?
- Oxygen: Treatment for carbon monoxide toxicity?
- Hydroxocobalamin: What is the cyanokit ingredient?
- Vitamin B12: Hydroxocobalamin will detoxify cyanide by binding with it to what?

##### Pearl #2

Carbon monoxide (CO) poisoning laboratory features and treatment.

- Carbon monoxide (CO) poisoning may manifest with non-specific symptoms, persistent neurologic symptoms or even as cardiac arrest.^11^
- Workup:
  ○ Pulse oximetry: Readings will be falsely normal
  ○ Co-oximetry: measures carboxyhemoglobin which is carbon monoxide bound to hemoglobin
  ○ ABG: PaO2 and % hemoglobin saturation unaffected but an “oxygen saturation gap,” which is the difference between the calculated oxygen saturation from a standard blood gas and the reading from a pulse oximeter may be present.^17^
  ○ Order a carboxyhemoglobin level. Subtract the carboxyhemoglobin level from the pulse oximetry level to determine true oxygen saturation.
    ■ Non-smokers: up to 1% normal
    ■ Smokers: 4–6% common
    ■ Any reading >10% = concern for significant exposure
- Treatment: Administer 100% O2. May use hyperbaric oxygen in very severe cases such as if there is loss of consciousness at the scene, new neurological deficits, mental status change, end-organ ischemia, or if patient is pregnant.^18^

##### Pearl #3

Cyanide (CN) poisoning laboratory features and treatment.^12^ Cyanide (CN) poisoning may manifest with variable symptoms from mydriasis and tachypnea to seizures and loss of consciousness.

- Workup:
  ○ Pulse oximetry: hypoxia
  ○ ABG: Anion gap metabolic acidosis with severely elevated lactate
- Treatment:
  ○ Administer100% O2 therapy
  ○ Administer hydroxocobalamin, with consideration for sodium thiosulfate (slower mechanism of action).
    ■ Note: The commercially available cyanokit contains hydroxocobalamin. Cyanide binds to hydroxocobalamin, forming cyanocobalamin (vitamin B12) which is renally excreted. The traditional cyanide antidotes include inhaled amyl nitrite, sodium nitrite, and sodium thiosulfate. Sodium nitrite forms methemoglobin from hemoglobin, for which cyanide has enormous affinity. Cyanide leaves the cytochrome, setting the mitochondria free, forming cyanmethemoglobin. This is transformed to thiocyanate by an enzyme (rhodanese) and renally excreted.

#### Activity 3

RESCUE, ALARM, CONFINE, EXTINGUISH/EVACUATE.

#### Activity 4

PULL, AIM, SQUEEZE, SWEEP.

##### Pearl #3/4

Actions to take in response to fire emergencies (R.A.C.E. and P.A.S.S. acronyms)

- R.A.C.E.
  ○ An acronym that hospital personnel use to remember their duties in case of fire
  ○ It stands for RESCUE, ALARM, CONFINE, EXTINGUISH/EVACUATE.
- P.A.S.S.
  ○ An acronym that hospital personnel use to remember their duties for discharging a fire extinguisher.
  ○ It stands for PULL, AIM, SQUEEZE, SWEEP.

#### Activity 5

A 53-year-old male presents to hospital after suffering a burn to his hand from his stove. On examination of his skin, it appears dry, pale and has a sluggish capillary refill. Some pale areas of his burn have no significant pain. How would you classify this burn? C: Deep Partial Thickness

A 5-year-old boy presents to the emergency department after burning his hand from spilled soup. He appears well. When you examine his skin, it appears erythematous with a brisk capillary refill. Three hours after his injury, you noticed that the area of erythema is starting to disappear. How would you classify this burn? A. Superficial Thickness

All should be performed on a burn except: C. apply silver sulfadiazine dressings to the wound to promote healing All are acceptable medications to take/use for burn complications except: D. Flammazine

#### Activity 6

[Fig f9-jetem-6-2-sg46]

**Figure f9-jetem-6-2-sg46:**
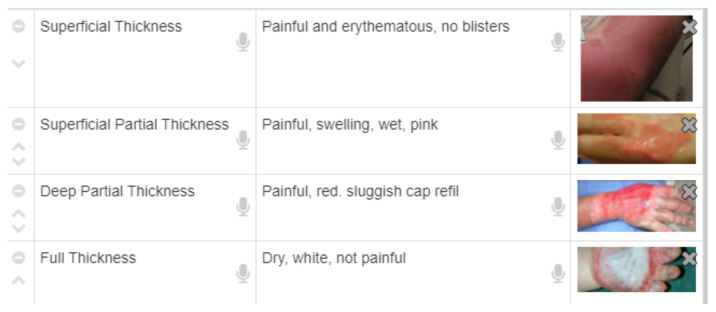


##### Pearl #5/6

Classification of burn injury according to depth, extent and severity based on established standards.^19^

**Figure:**
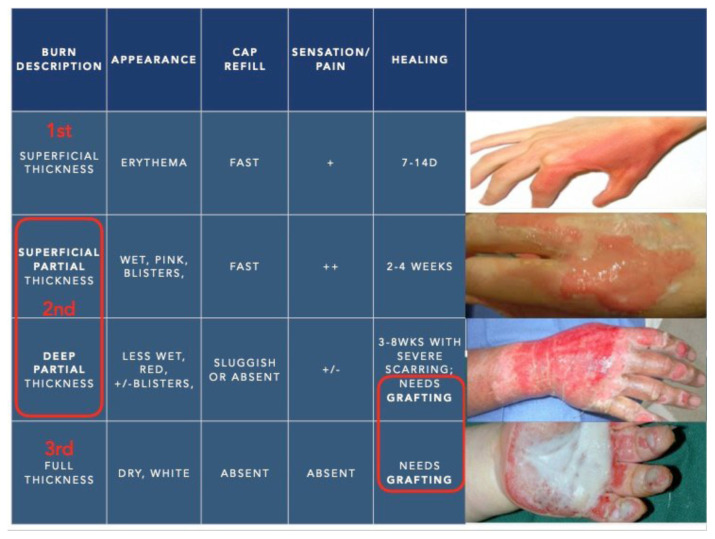


#### Activity 7

Indications for consultation to burn center: Full thickness burn, Partial thickness burns > 10%, Burns to hands, Emotional or social need, burn to genitalia

Non-indications for burn center: Superficial burn > 50%, Burn to chest wall, Low voltage electrical injury, all pediatric patients

##### Pearl #7

Indications for referral to burn center

American Burn Association. Burn center referral criteria. http://www.ameriburn.org/BurnCenterReferralCriteria.pdf. Accessed July 30, 2013.

#### Activity 8

What is the urine output goal in cc/hr for a 20kg child (input the answer in the lock)? 20.

##### Pearl #8

Fluid resuscitation in burn patients.^13^ In order to determine the volume of fluid resuscitation required for a burn patient, the Rule of Nines for adults and the Lund and Browder chart for children should be utilized.

- Remember: do not include first degree burns in the calculation of % TBSA.
  ○ 2–4mL x kg body weight x % TBSA burn = volume of Lactated Ringer’s required for adult resuscitation (formula adjusted to 3–4mL x kg body weight x % TBSA burn for pediatric patients).
- Half of the total resuscitation volume is given over the first 8 hours, with administration of the remaining half titrated to patient response (urine output goal of 0.5mL/kg/hr for adults and 1mL/kg/hr for children).
- All resuscitation measures should be guided by perfusion pressure and urine output:
  ○ Target a MAP of 60 mmHg, and urine output of 0.5–1.0ml/kg/hr for adults and 1mL/kg/h for pediatric patients.

### Results and tips for successful implementation

A total of three Virtual Escape Room sessions were held at two academic institutions during the month of April 2020, one during an emergency medicine (EM) didactic, one during family medicine (FM) didactics, and the last for a joint family medicine and emergency-family medicine (EM/FM) didactic session. Each session was led by one instructor who conducted the introductory session, small group facilitation, debriefing and content overview. There was a total of 63 participants composed of residents (24 EM, 29 FM, 4 EM/FM), advanced practice practitioner trainees (2 EM), and faculty participants (4 FMP), all of whom participated as equal team members within the group. Participants all signed into a Zoom video conferencing room, and attendance was tracked through Zoom registration so that room assignments could be randomized. The instructor randomly divided attendees into groups of four to six members during each didactic session with an attempt to assure an equal distribution of learner experience based on level of training. Four to six members was an ideal group size, allowing for small group interaction. Each group should be assigned a team lead, with the role of sharing their screen and inputting team answers into each learning activity. The team lead accessed the Deck.Toys link (deck.toys/virtualescaperoom) and screen-shared the activity for all team members to see and engage. Other team members were not provided with predetermined roles or task designations. All participants were separated into 4-to-6-member small groups via Zoom breakout rooms. The instructor entered each room periodically to answer urgent questions and observe team behaviors without directly participating in the activity or answering topic content-related or knowledge-based questions. Ideally, to reduce the duties of the instructor and be more effective, each team would have a faculty volunteer to perform these tasks. Question format varied from multiple choice questions that did not have to be answered correctly but informed residents of correct answers to cross-word puzzles and matching games in order to mimic the in-person escape room experience and encourage brainstorming, thinking out loud, and teamwork (see Figure 5). During the challenge in their Zoom breakout rooms, participants were allowed to use any available resources (websites, textbooks, mobile applications), other than the instructor, to answer the questions. Each team was given 15 minutes to complete the entire activity. After 15 minutes, the team that either escaped the room the fastest or progressed the farthest was announced. Although each session had at least one team complete the activity in the allotted time, a majority of teams did not complete the activity in time due to various reasons including lack of technical expertise, lack of team interaction, difficulty figuring out task puzzles, and knowledge deficits. To assure a higher completion rate, instructors can consider increasing activity completion time to 20–25 minutes or providing reading materials for pre-session preparation. After announcing the winner of the activity, the instructor conducted a 5-minute debriefing session followed by a 30-minute overview of the content covered (refer to PowerPoint attached).

### Evaluation

At the end of the activity, a 17-item survey using Likert-scale questions was embedded in order to obtain feedback regarding satisfaction, engagement, learning, and medical competency. We adapted pre-existing survey instruments to assess resident satisfaction, motivation, learning, and skills. We used items adapted from Kinio et al, Meterissian et al, and Jambhekar et al.^6^–^8^ The overall response rate for the post-event survey was 18 of 63 participants (28.6%), which may have been due to the dependence of scanning the QR code or copying the survey link in order to complete the evaluation. Seventeen out of eighteen respondents had never experienced a virtual escape room. Survey and responses are available in supplemental material (Table 1).

#### Satisfaction

A majority (16/18 = 88.9%) of participants enjoyed the virtual escape room, with 94.4% (17 out of 18) rating the activity as fun. Five out of 18 (38.9%) rated the activity as stressful. None of the participants preferred traditional didactics over the virtual escape room activity, and 72.2% were either just as or equally as satisfied with virtual compared to in-person escape rooms.

#### Engagement

Engagement was high, with all categories receiving the nearly 100%. They found the challenges interesting, engaging, and interactive (94.4%).

#### Learning

As a learning platform, participants felt that the game was helpful in increasing (88.9%) and retaining (72.2%) clinical information. Learners either agreed or strongly agreed (88.89%) that the format helped them identify knowledge gaps. Most of the participants (94.4%) gained new knowledge while 77.8% felt that they will apply what they’ve learned in the future.

#### Competencies

Collaboration was the most encouraged skill during the activity, with 94.4% either agreeing or strongly agreeing that the activity encouraged the skill’s use. Communication skills were also encouraged (88.9%). Greater than two thirds of participants agreed that it encouraged task-switching and leadership skills.

## Figures and Tables

**Table 1:** Advanced Burn Life Support (ABLS) burn center referral criteria

Partial-thickness burns >10% of total body surface area Burns on face, hands, feet, genitalia, perineum, or major joints Third-degree burns in any age group Electrical burns, including lightning burns Chemical burns Inhalation burns Burn injury in patients with preexisting medical conditions that could complicate management, prolong recovery, or affect mortality Any patient with burns and concomitant trauma (eg, fractures) in which the burn injury poses the greater risk of morbidity or mortality. If trauma poses the greater immediate risk, the patient may be stabilized in a trauma center before transfer to a burn unit. Physician judgment in these cases should reflect the regional medical control plan and triage protocols. Children with burns in hospitals without qualified personnel or equipment for pediatric care Burn injury in patients who will require social, emotional, or rehabilitative interventions

Adapted from American Burn Association.^3^

## SMALL GROUP MATERIALS

### Appendix A Virtual Escape Room Burn and Inhalation Injuries

Please see associated PowerPoint file
