# Supplementary material for: Let’s Escape Didactics: Virtual Escape Room as a Didactic Modality in Residency
Source: J Educ Teach Emerg Med. 2021 Apr 19;6(2):SG46–56. doi: 10.21980/J8CH2X (PMC10332780; doi:10.21980/J8CH2X)
Supplement: Supplementary file 1 — Please see associated PowerPoint file [file jetem-6-2-sg46-appendixA.pptx]

## Slide 1
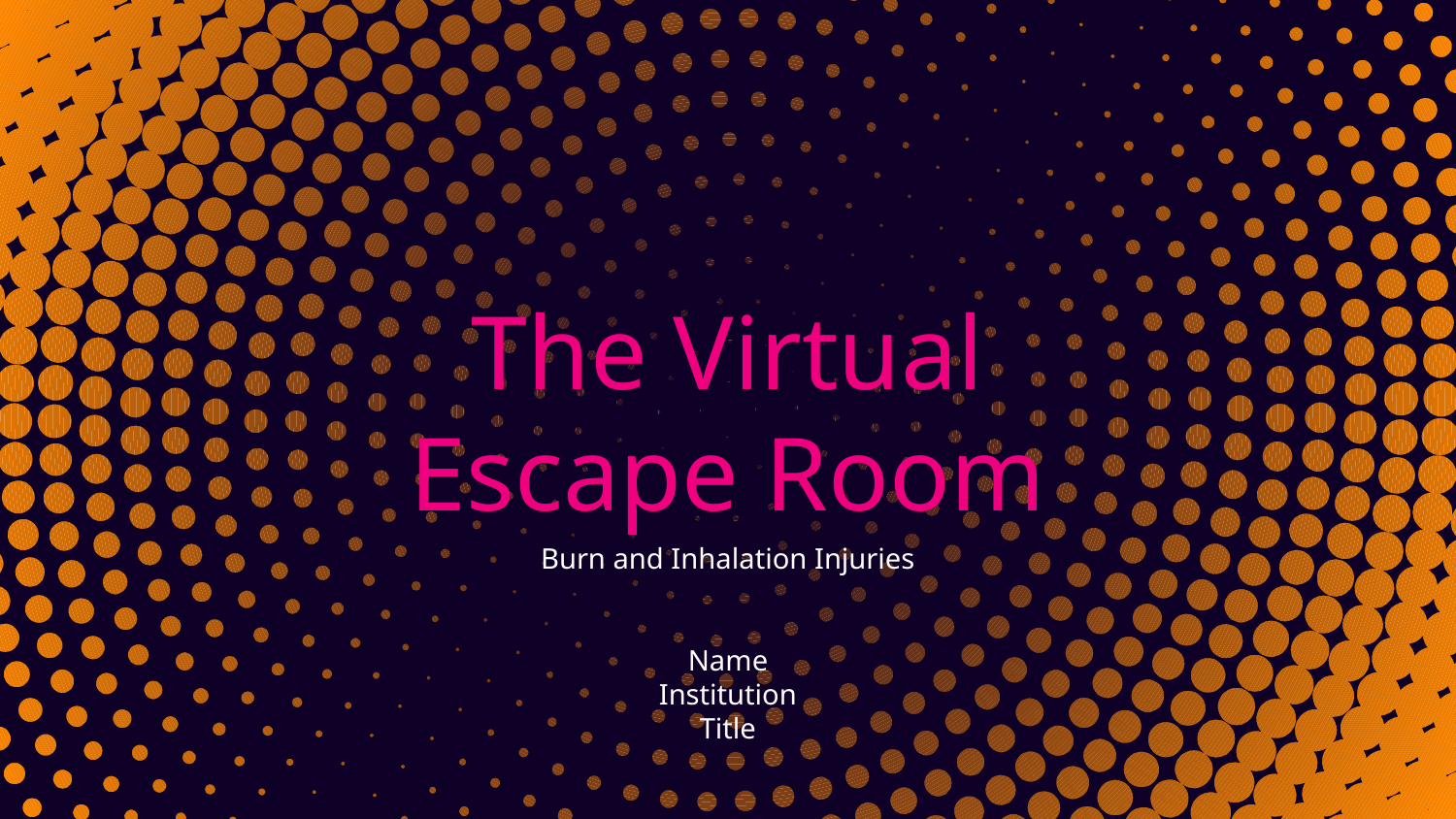

# The Virtual Escape Room
Burn and Inhalation Injuries
Name
Institution
Title

## Slide 2
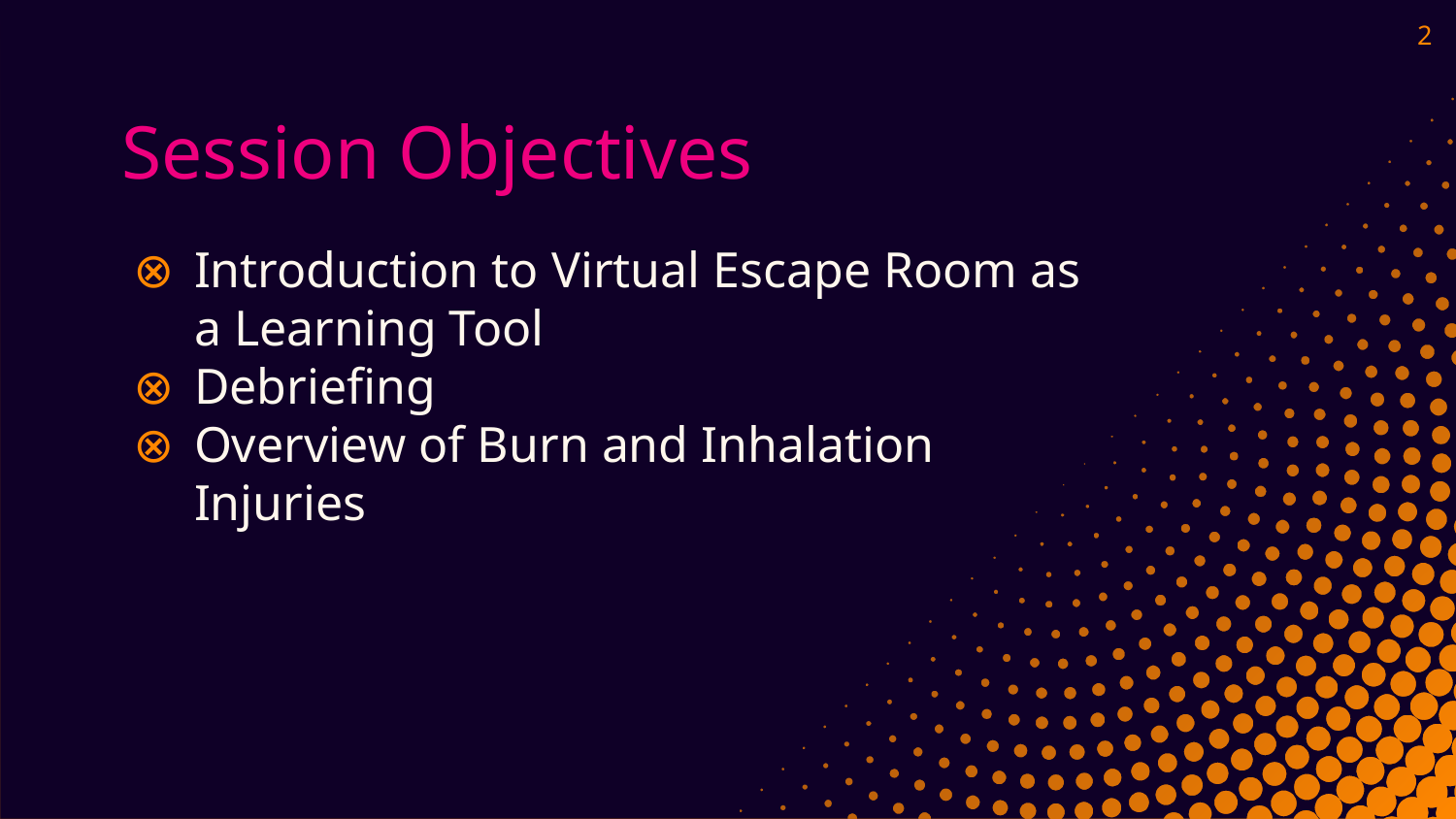

2
# Session Objectives
Introduction to Virtual Escape Room as a Learning Tool
Debriefing
Overview of Burn and Inhalation Injuries

## Slide 3
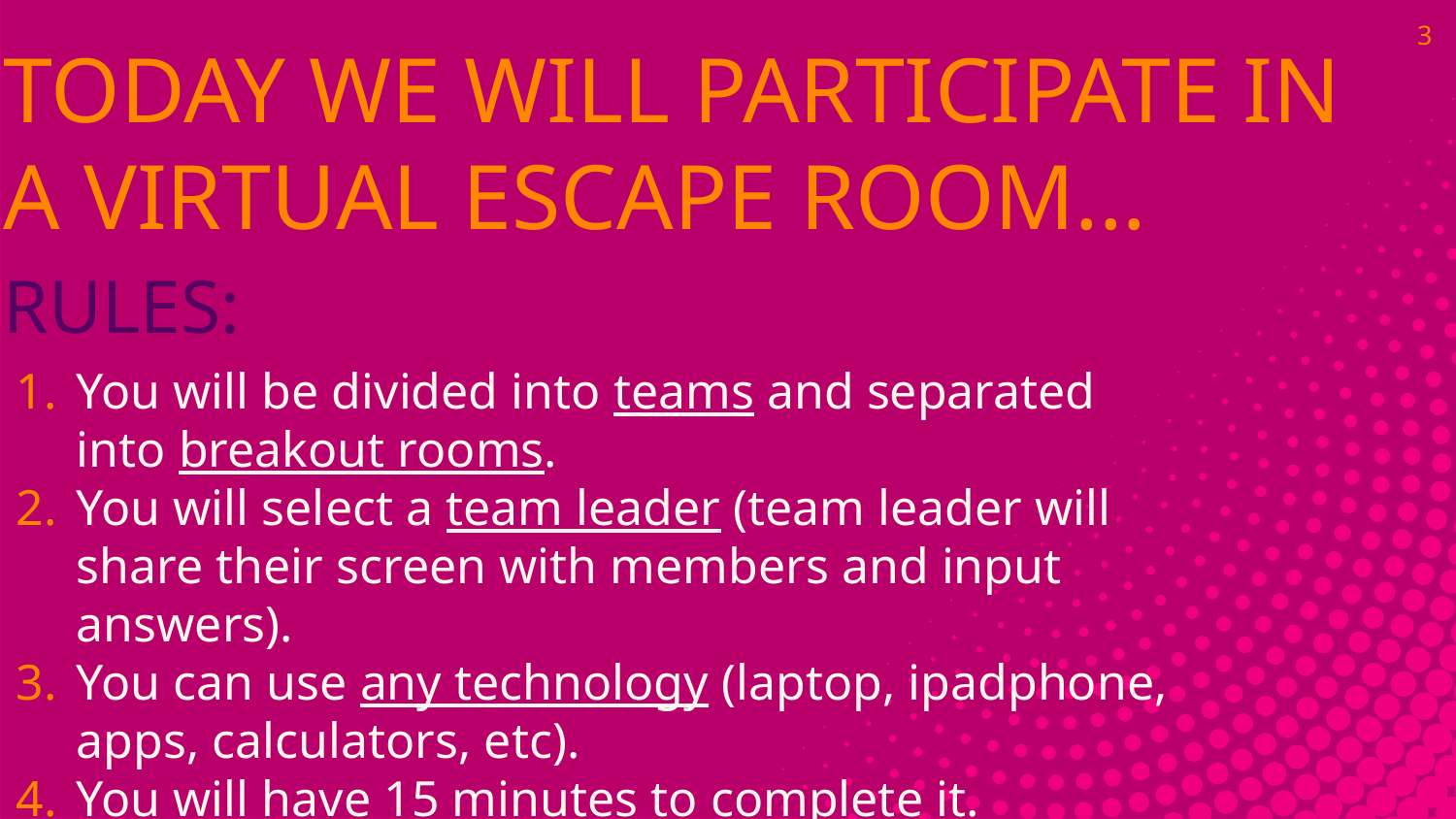

3
TODAY WE WILL PARTICIPATE IN A VIRTUAL ESCAPE ROOM...
RULES:
You will be divided into teams and separated into breakout rooms.
You will select a team leader (team leader will share their screen with members and input answers).
You can use any technology (laptop, ipadphone, apps, calculators, etc).
You will have 15 minutes to complete it.

## Slide 4
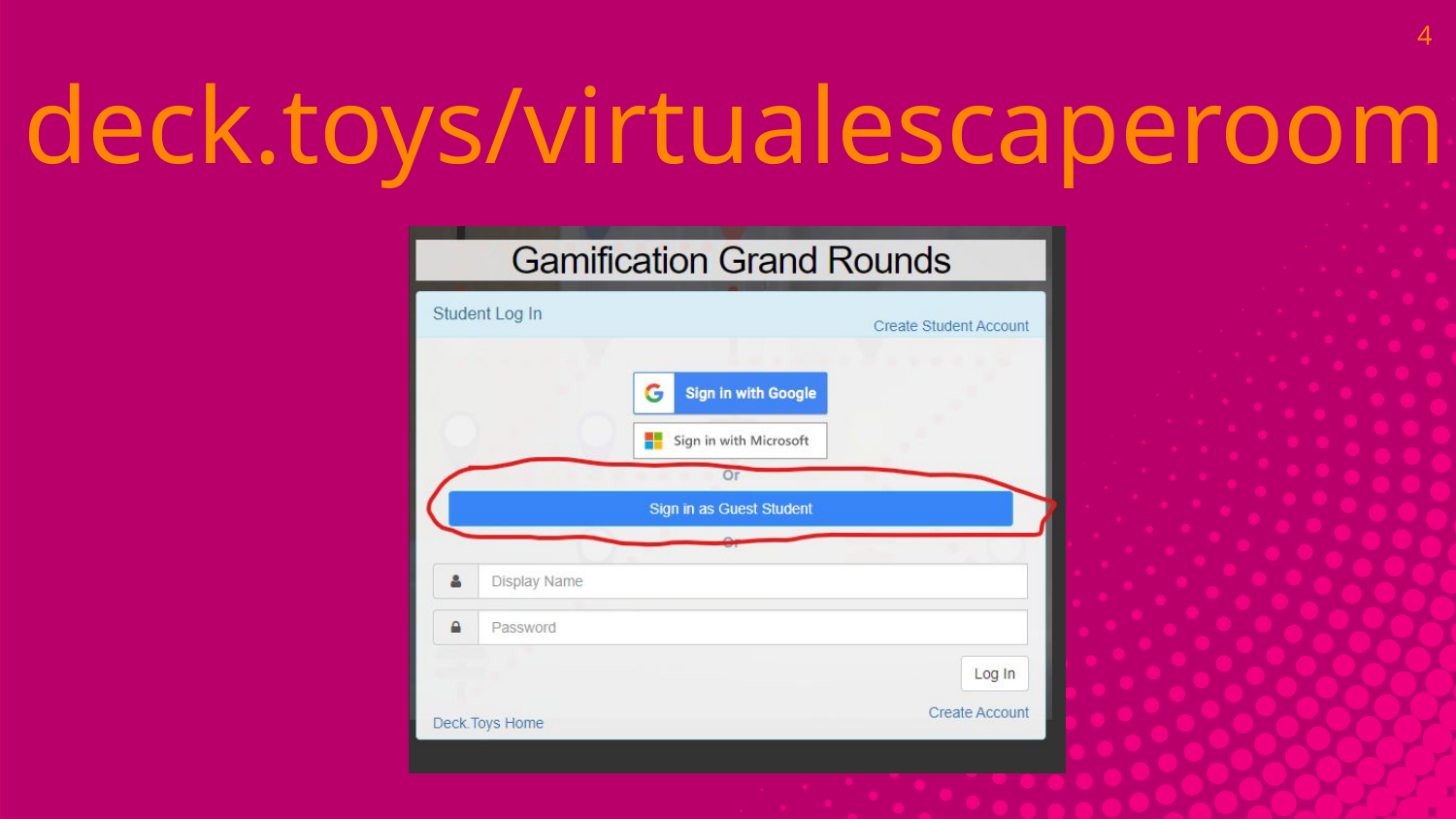

deck.toys/virtualescaperoom
4

## Slide 5
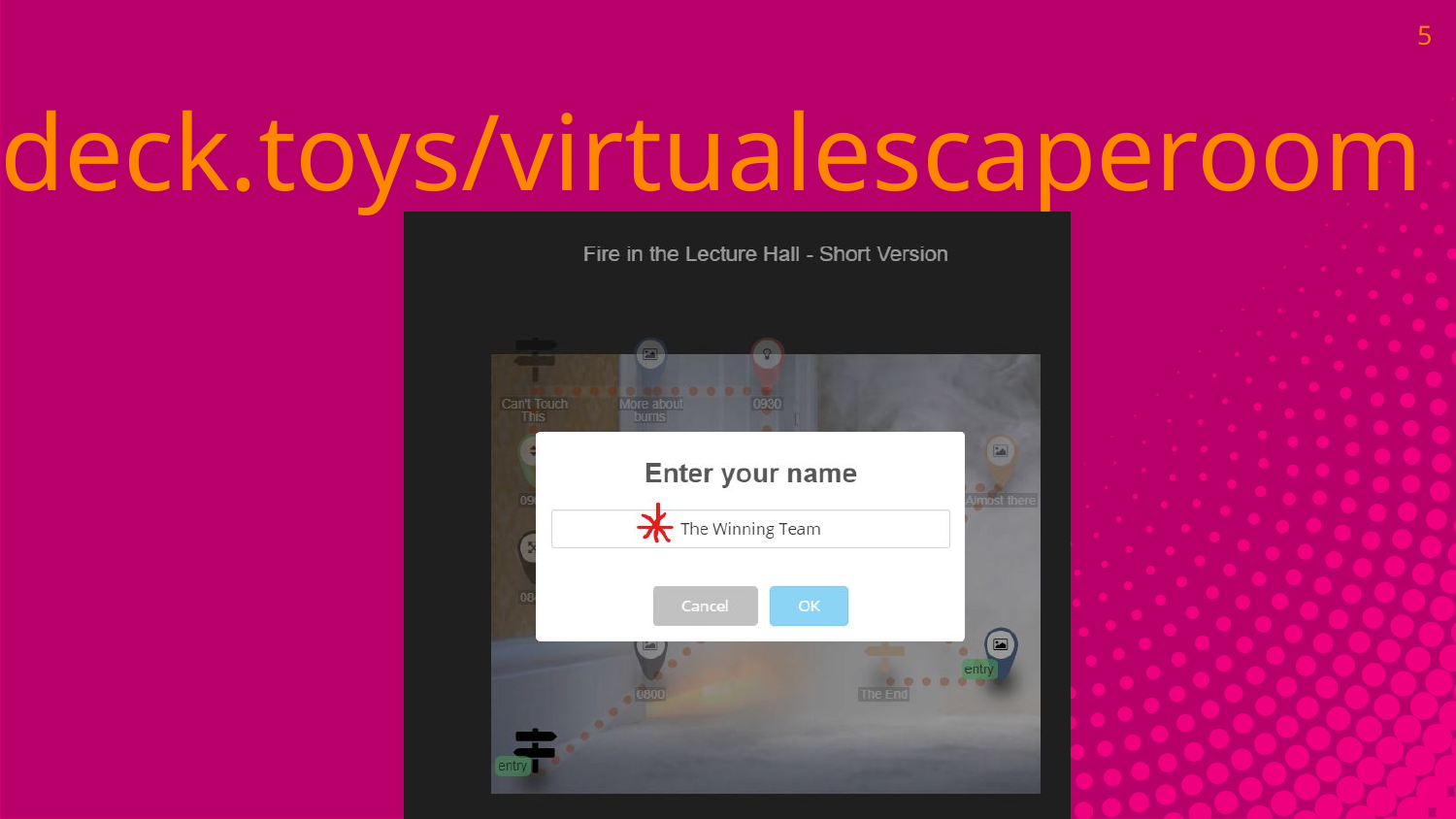

5
deck.toys/virtualescaperoom

## Slide 6
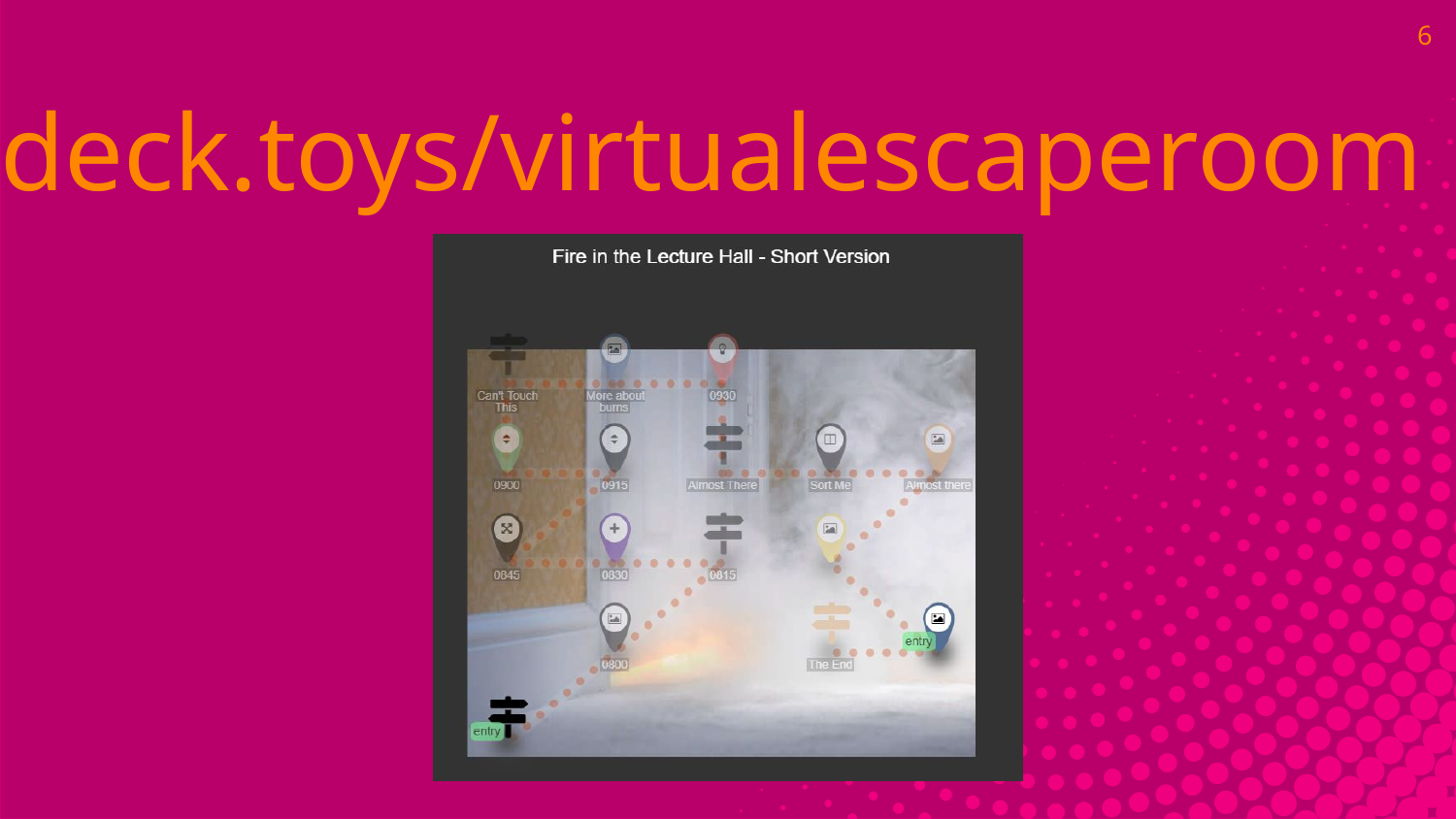

6
deck.toys/virtualescaperoom

## Slide 7
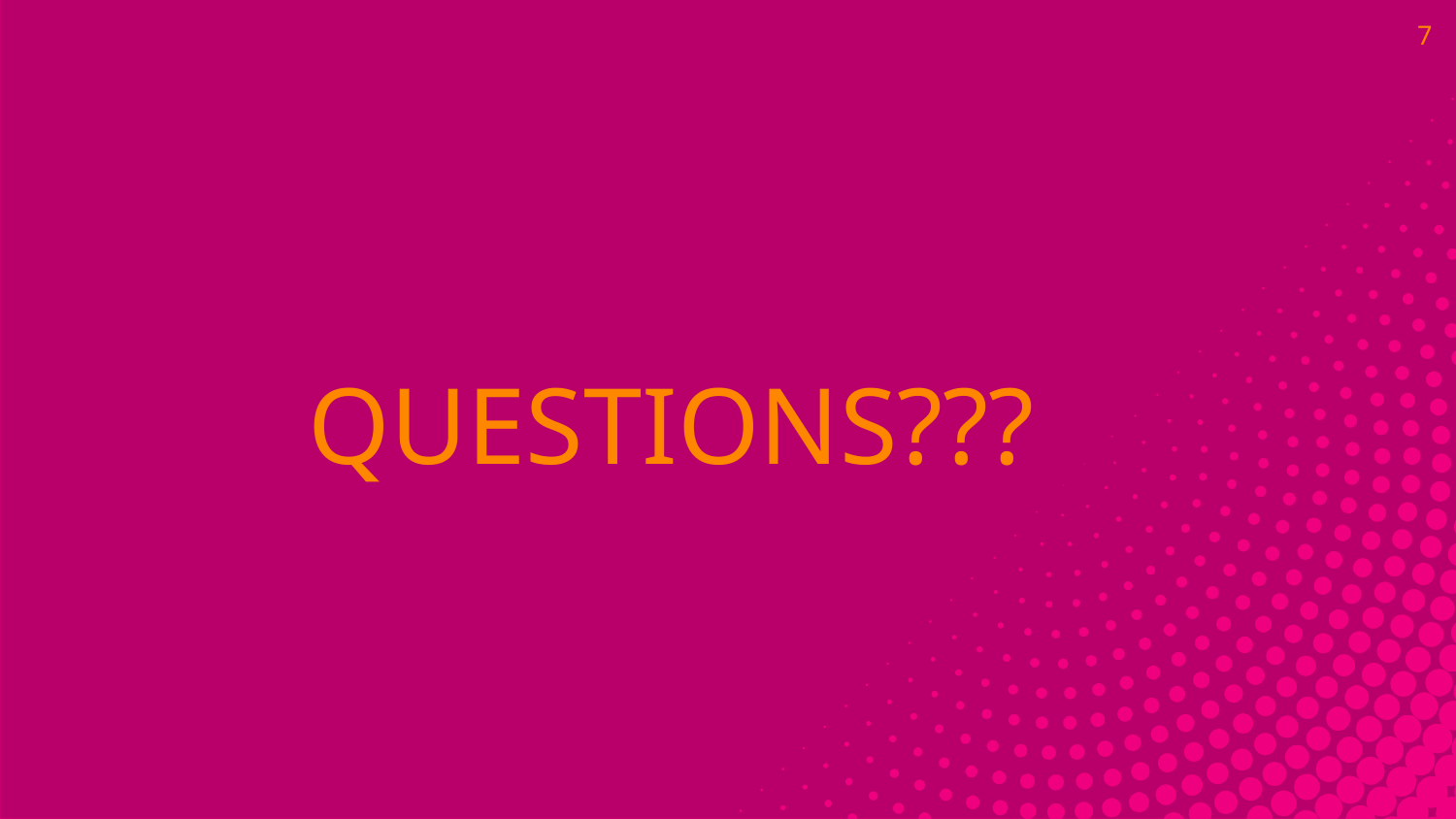

7
QUESTIONS???

## Slide 8
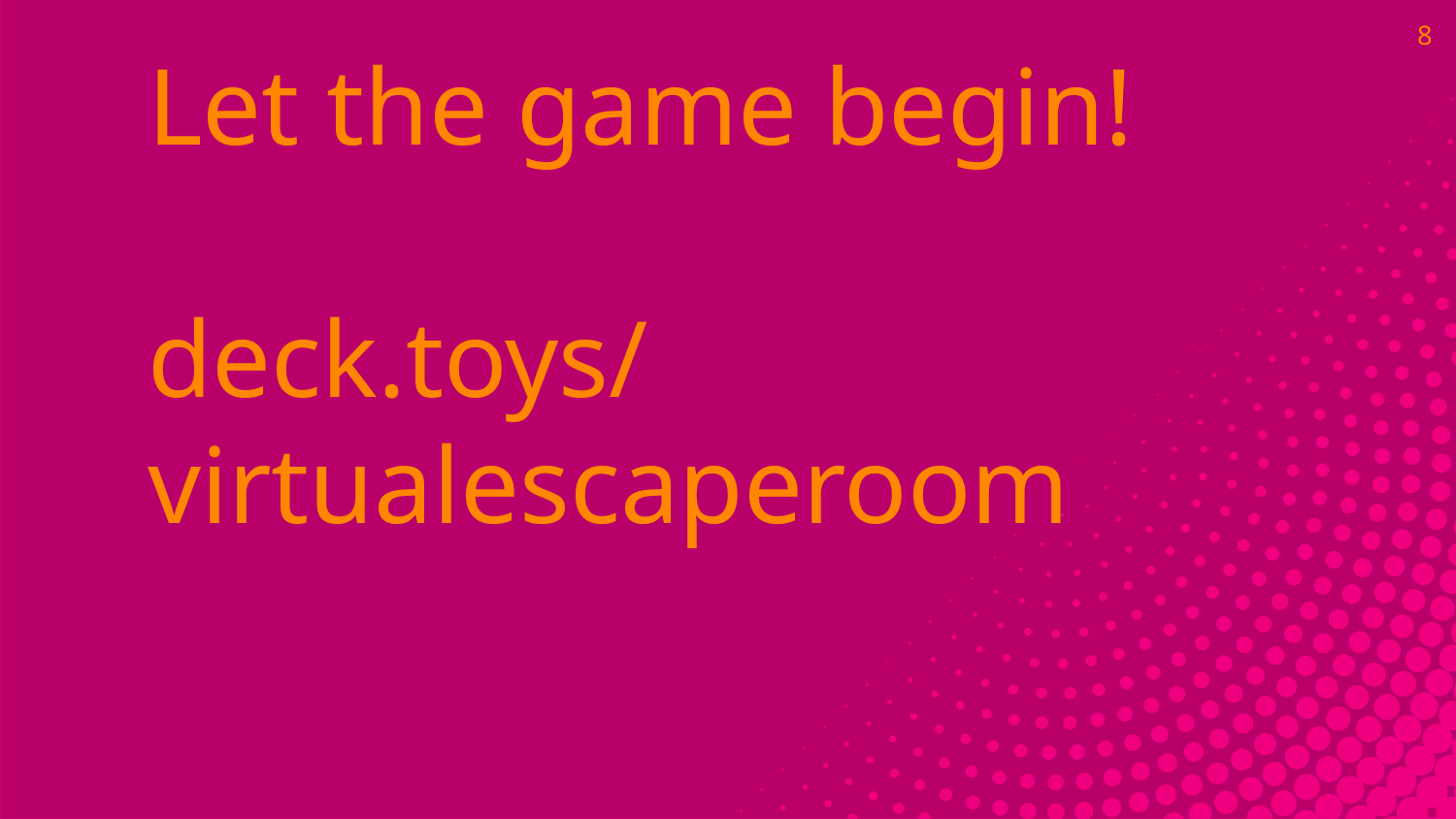

8
Let the game begin!
deck.toys/virtualescaperoom

## Slide 9
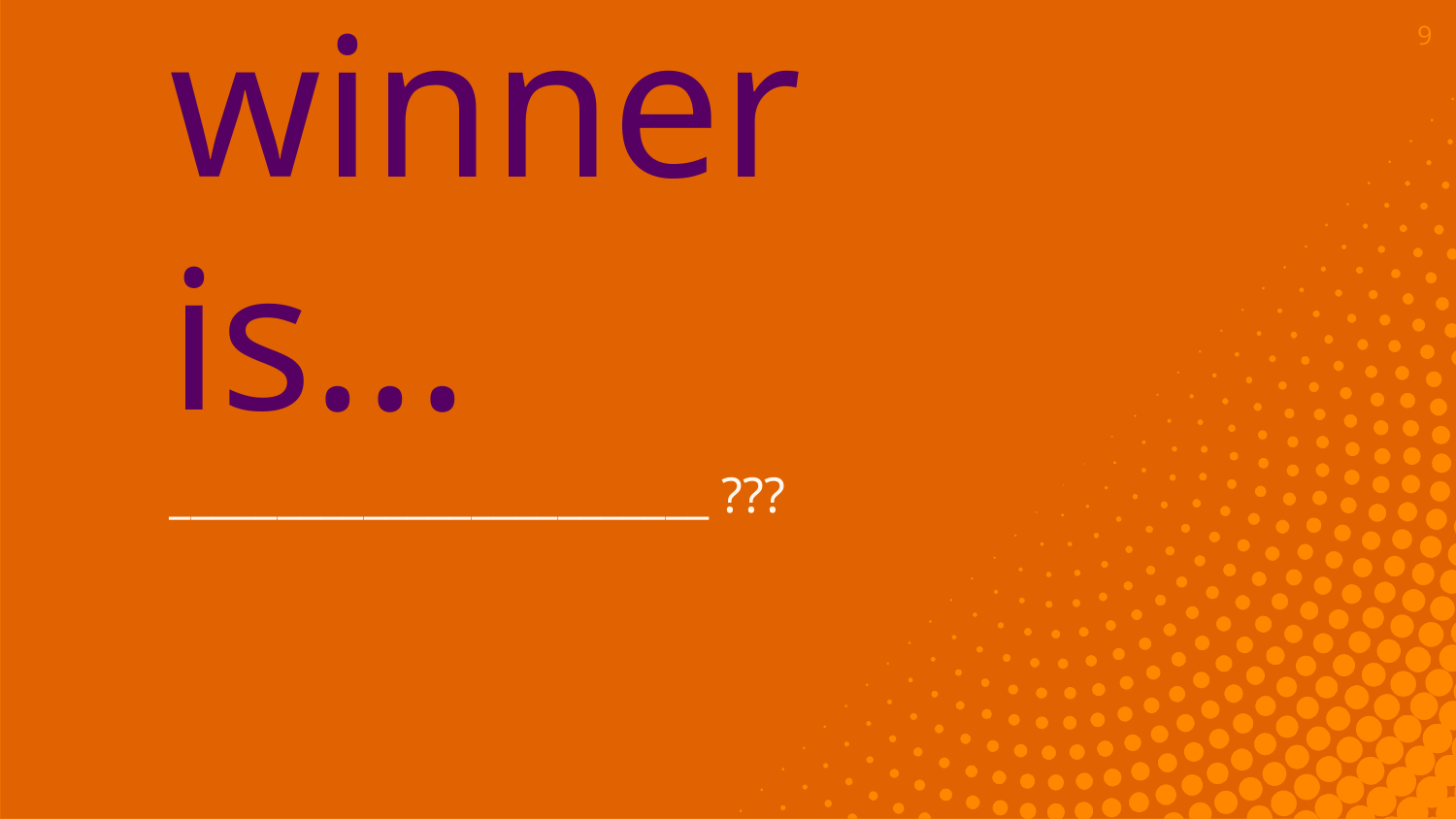

9
And the winner is...
_________________________ ???

## Slide 10
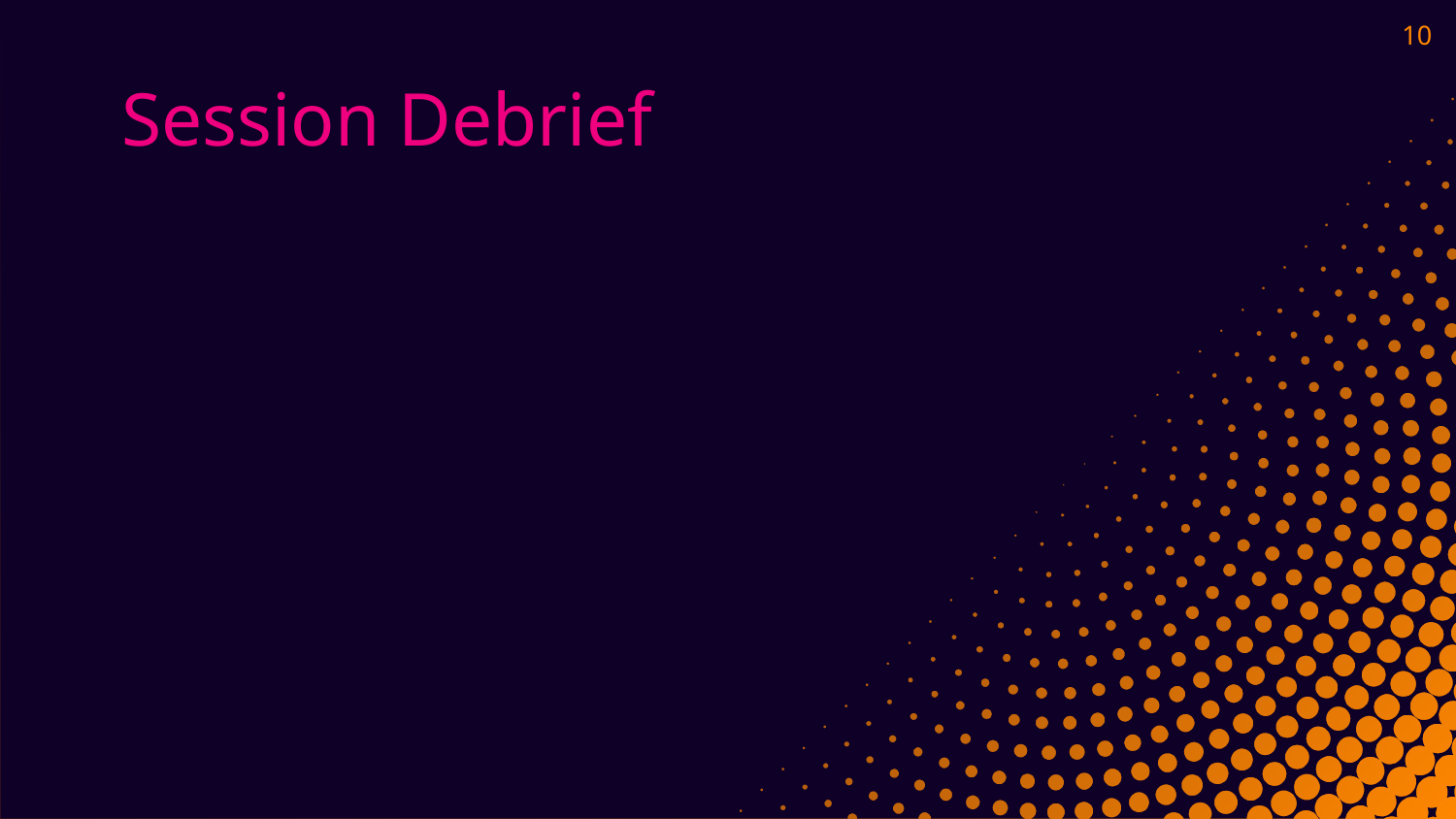

10
# Session Debrief

## Slide 11
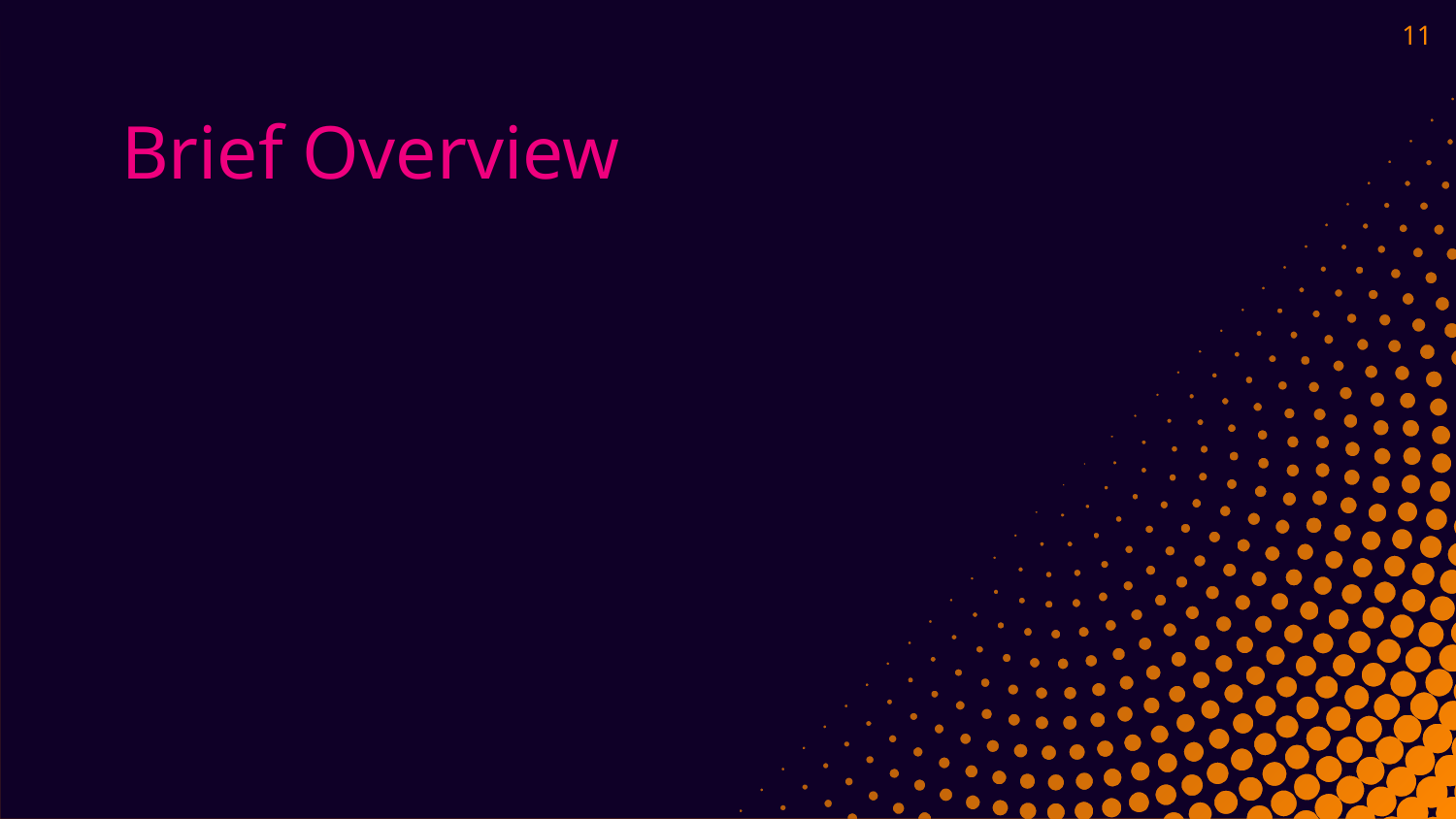

11
# Brief Overview

## Slide 12
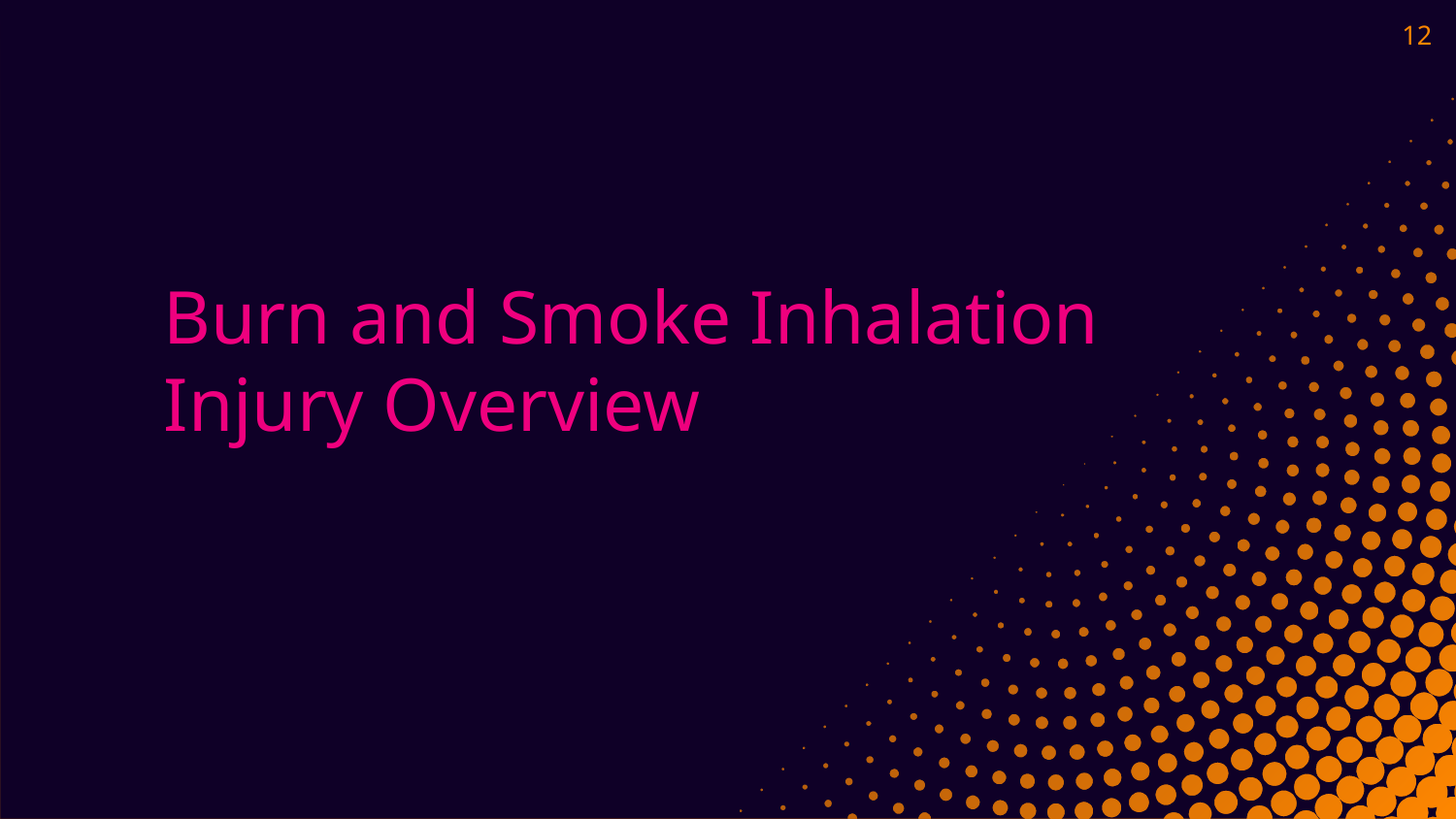

12
# Burn and Smoke Inhalation Injury Overview

## Slide 13
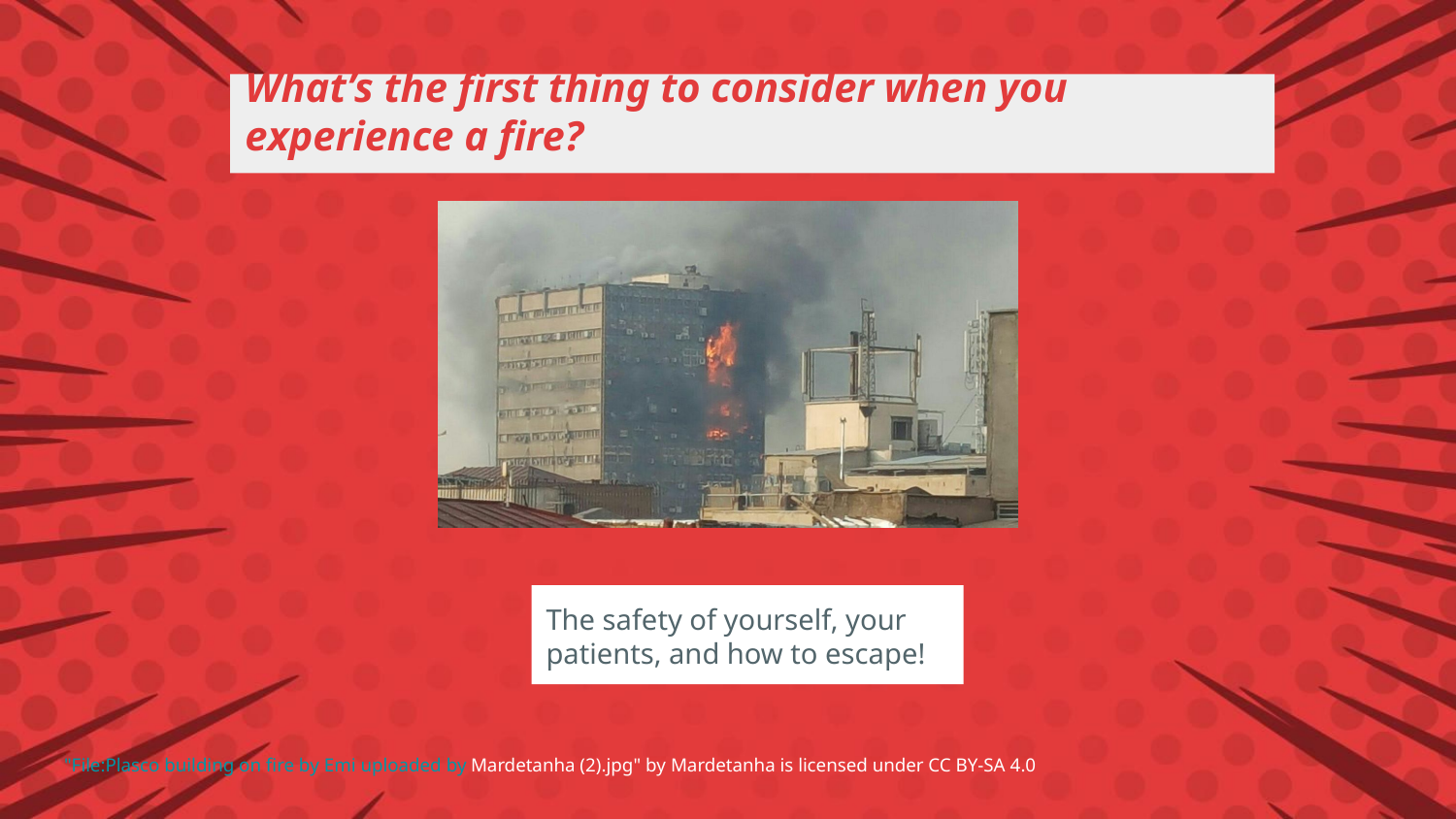

What’s the first thing to consider when you experience a fire?
The safety of yourself, your patients, and how to escape!
"File:Plasco building on fire by Emi uploaded by Mardetanha (2).jpg" by Mardetanha is licensed under CC BY-SA 4.0

## Slide 14
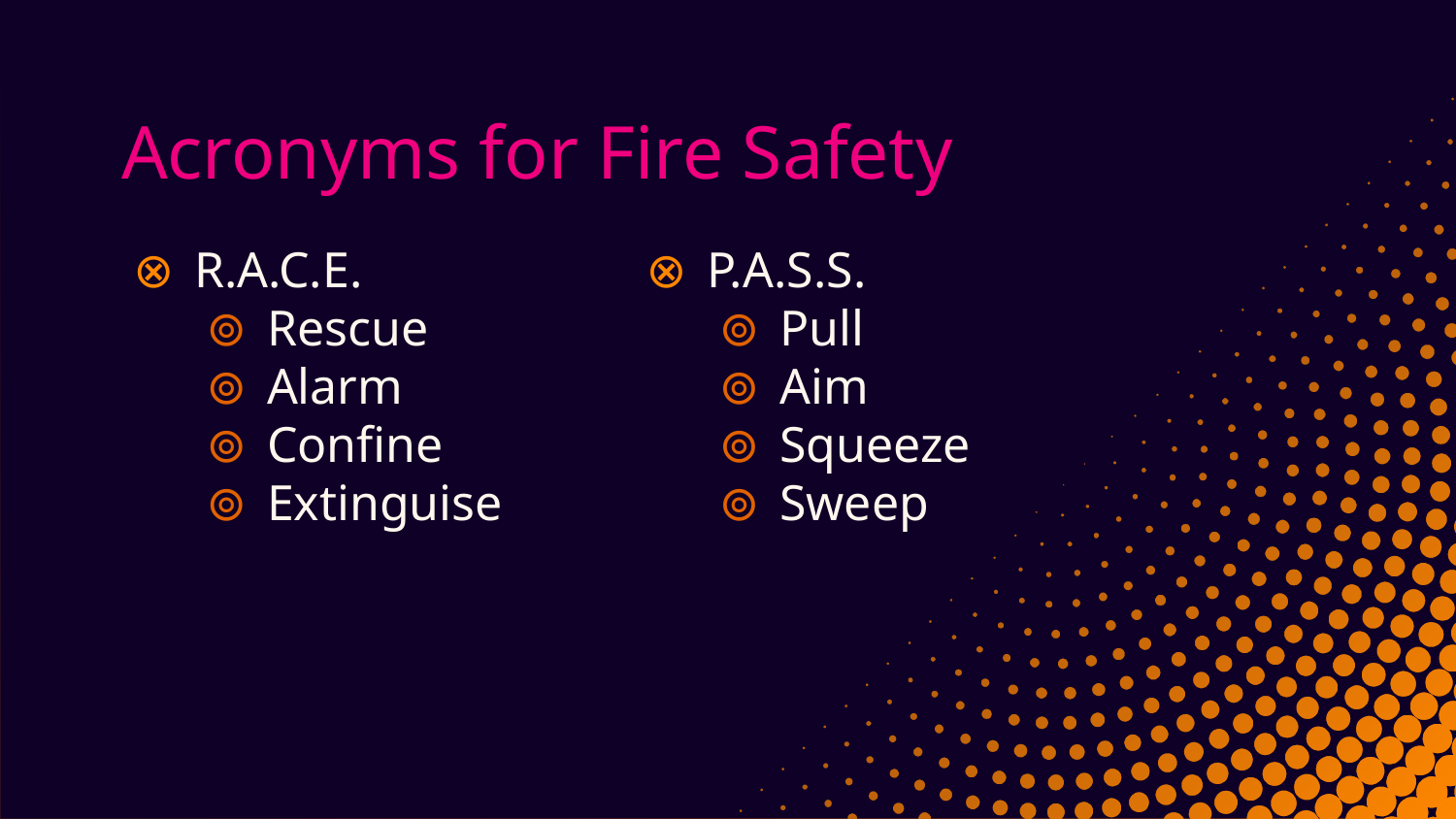

# Acronyms for Fire Safety
R.A.C.E.
Rescue
Alarm
Confine
Extinguise
P.A.S.S.
Pull
Aim
Squeeze
Sweep

## Slide 15
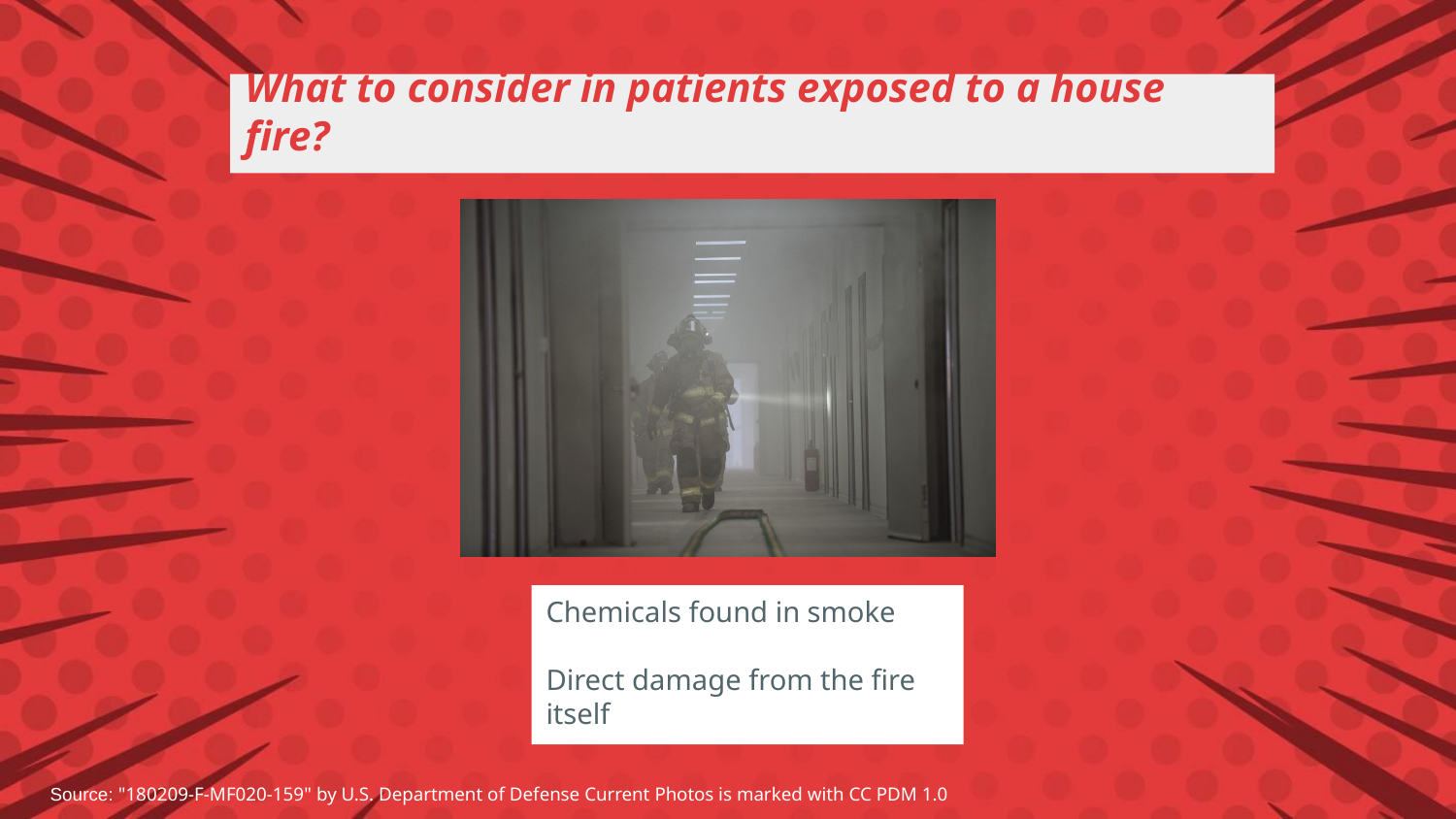

What to consider in patients exposed to a house fire?
Chemicals found in smoke
Direct damage from the fire itself
Source: "180209-F-MF020-159" by U.S. Department of Defense Current Photos is marked with CC PDM 1.0

## Slide 16
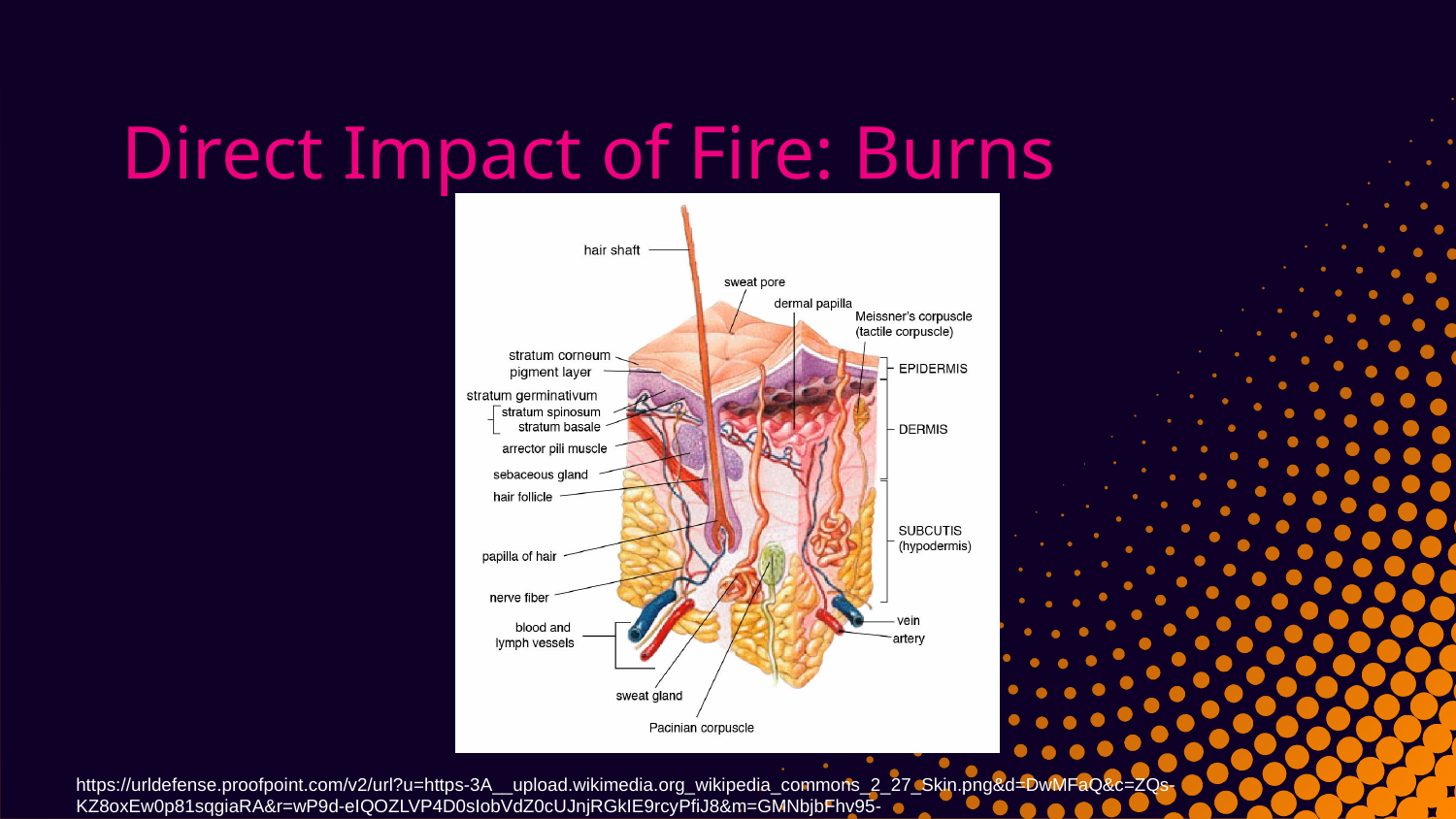

# Direct Impact of Fire: Burns
https://urldefense.proofpoint.com/v2/url?u=https-3A__upload.wikimedia.org_wikipedia_commons_2_27_Skin.png&d=DwMFaQ&c=ZQs-KZ8oxEw0p81sqgiaRA&r=wP9d-eIQOZLVP4D0sIobVdZ0cUJnjRGkIE9rcyPfiJ8&m=GMNbjbFhv95-WyeYhDG6tqGHIDSttu93wJIzyPK2jk4&s=GbvZ0joxV8b1h5vFTOnMCIKhDei1CgyQj6l1_92MS6w&e=

## Slide 17
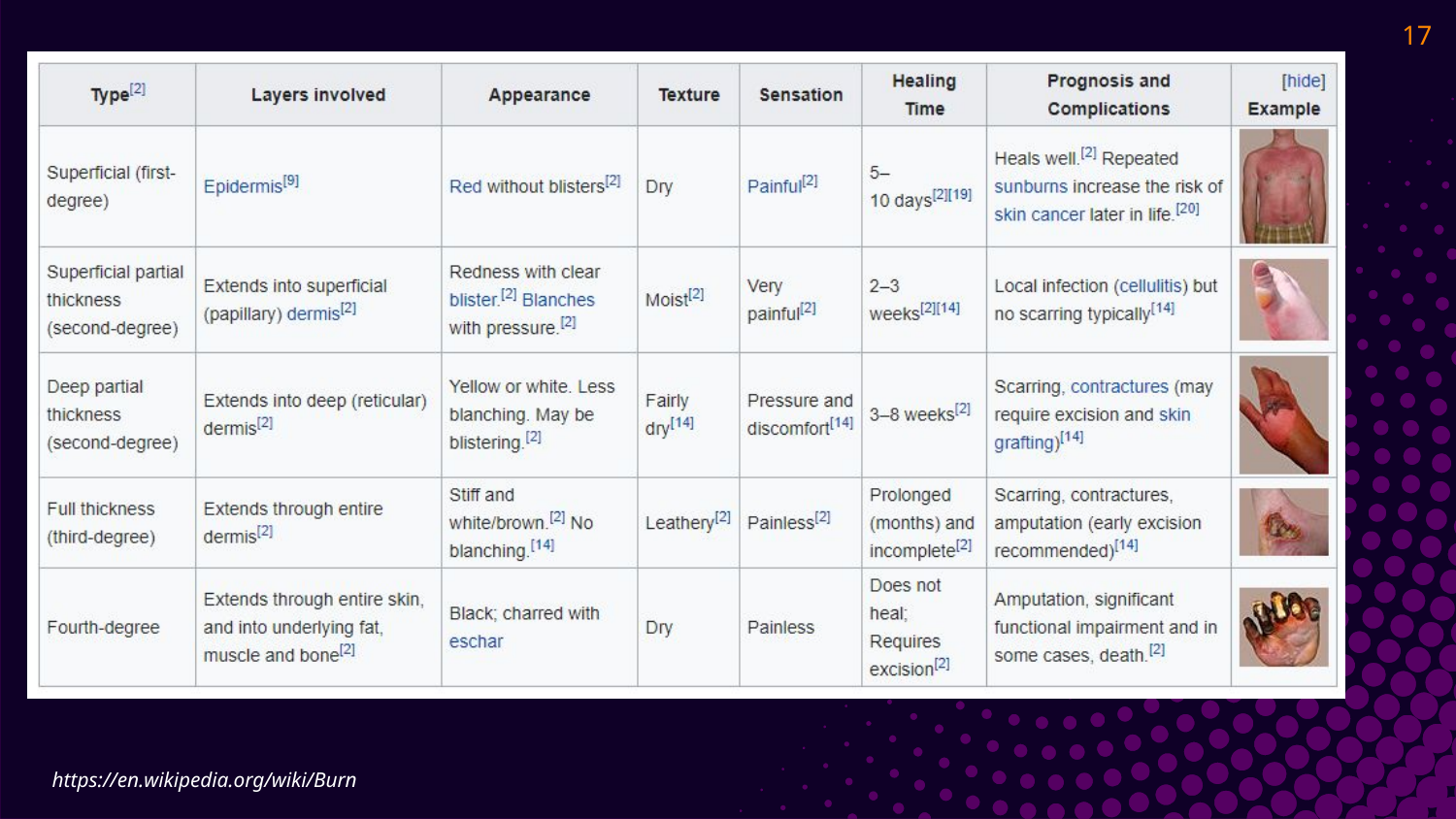

17
https://en.wikipedia.org/wiki/Burn

## Slide 18
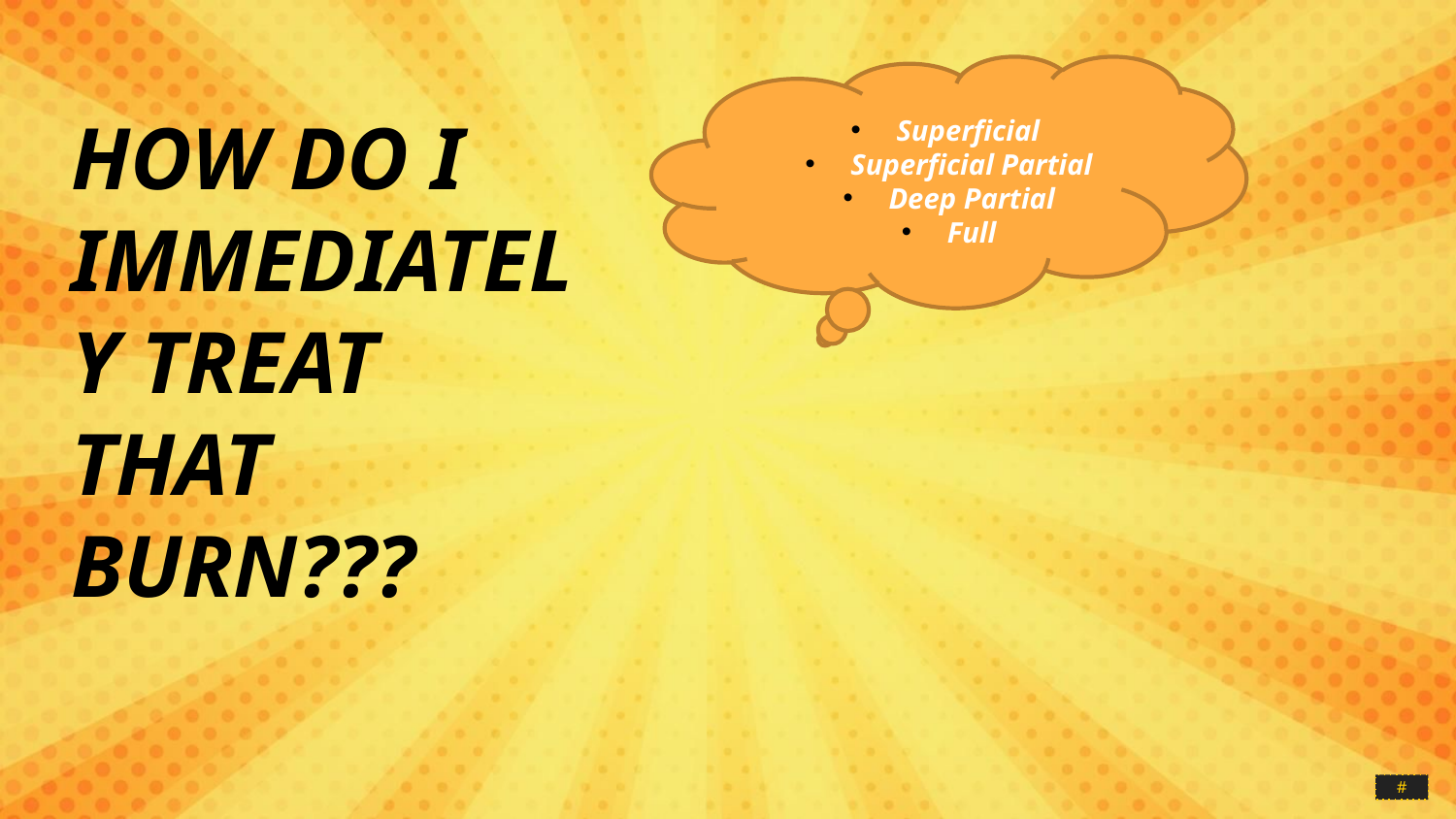

HOW DO I IMMEDIATELY TREAT THAT BURN???
Superficial
Superficial Partial
Deep Partial
Full
#

## Slide 19
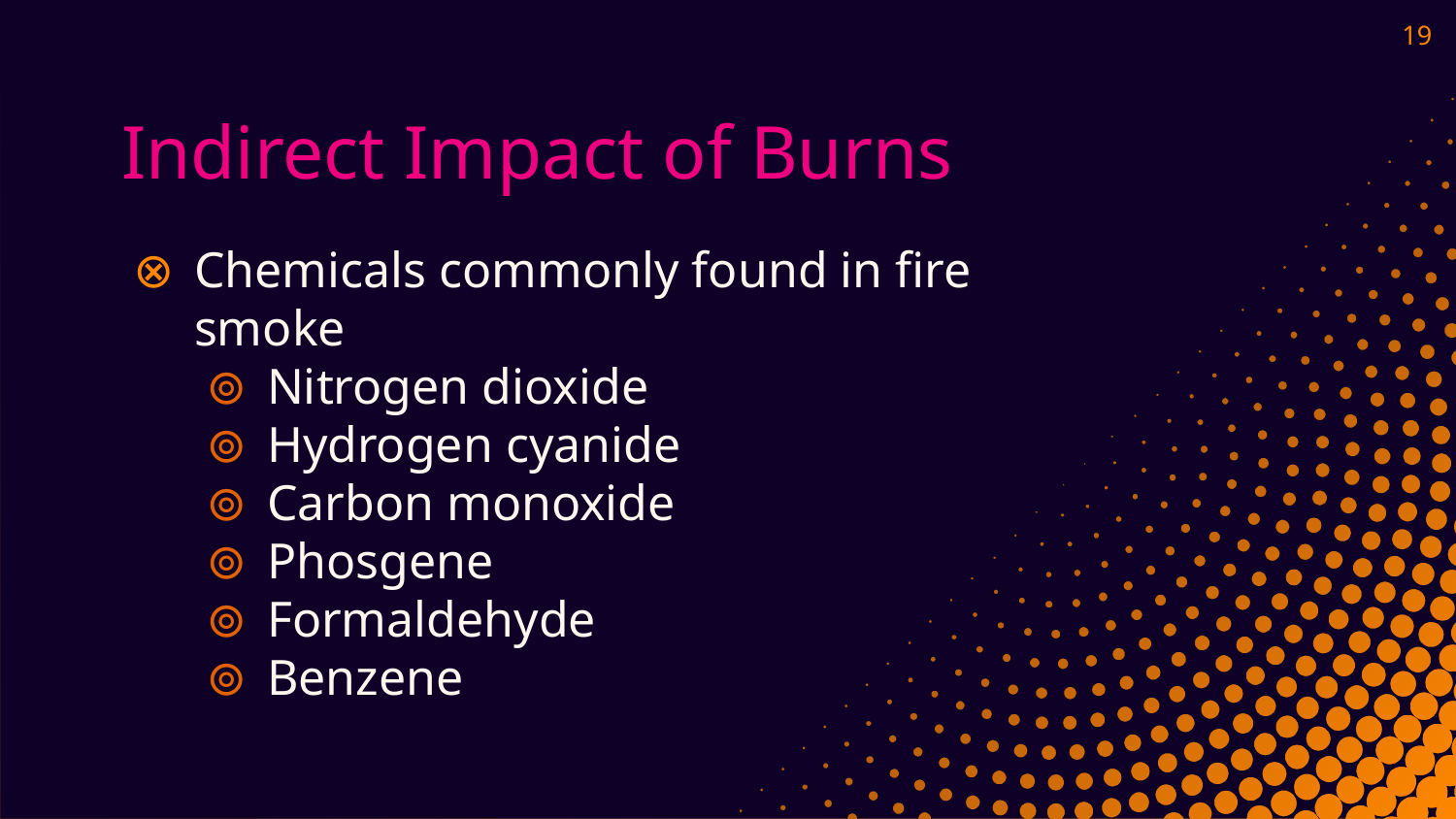

19
# Indirect Impact of Burns
Chemicals commonly found in fire smoke
Nitrogen dioxide
Hydrogen cyanide
Carbon monoxide
Phosgene
Formaldehyde
Benzene

## Slide 20
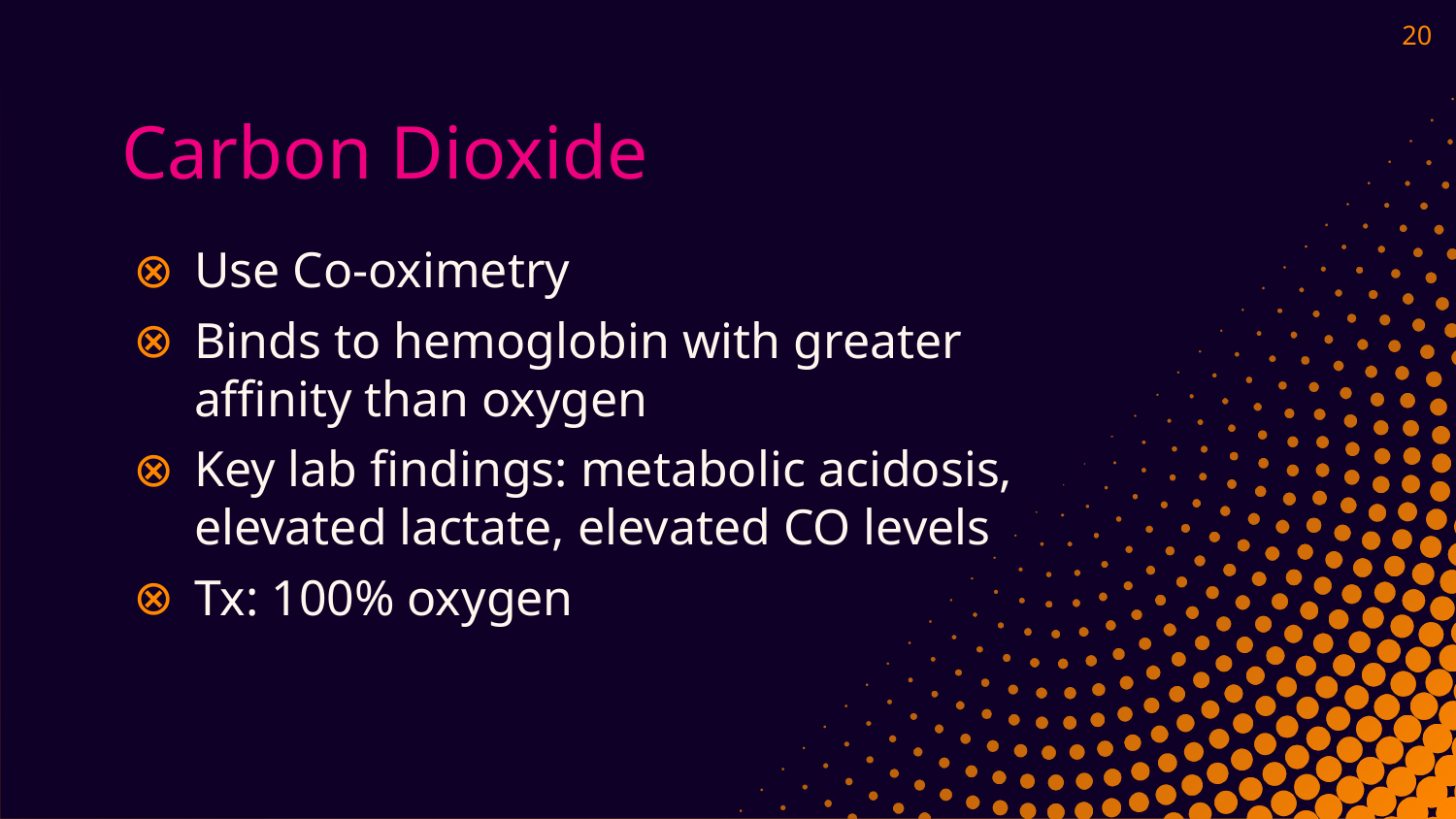

20
# Carbon Dioxide
Use Co-oximetry
Binds to hemoglobin with greater affinity than oxygen
Key lab findings: metabolic acidosis, elevated lactate, elevated CO levels
Tx: 100% oxygen

## Slide 21
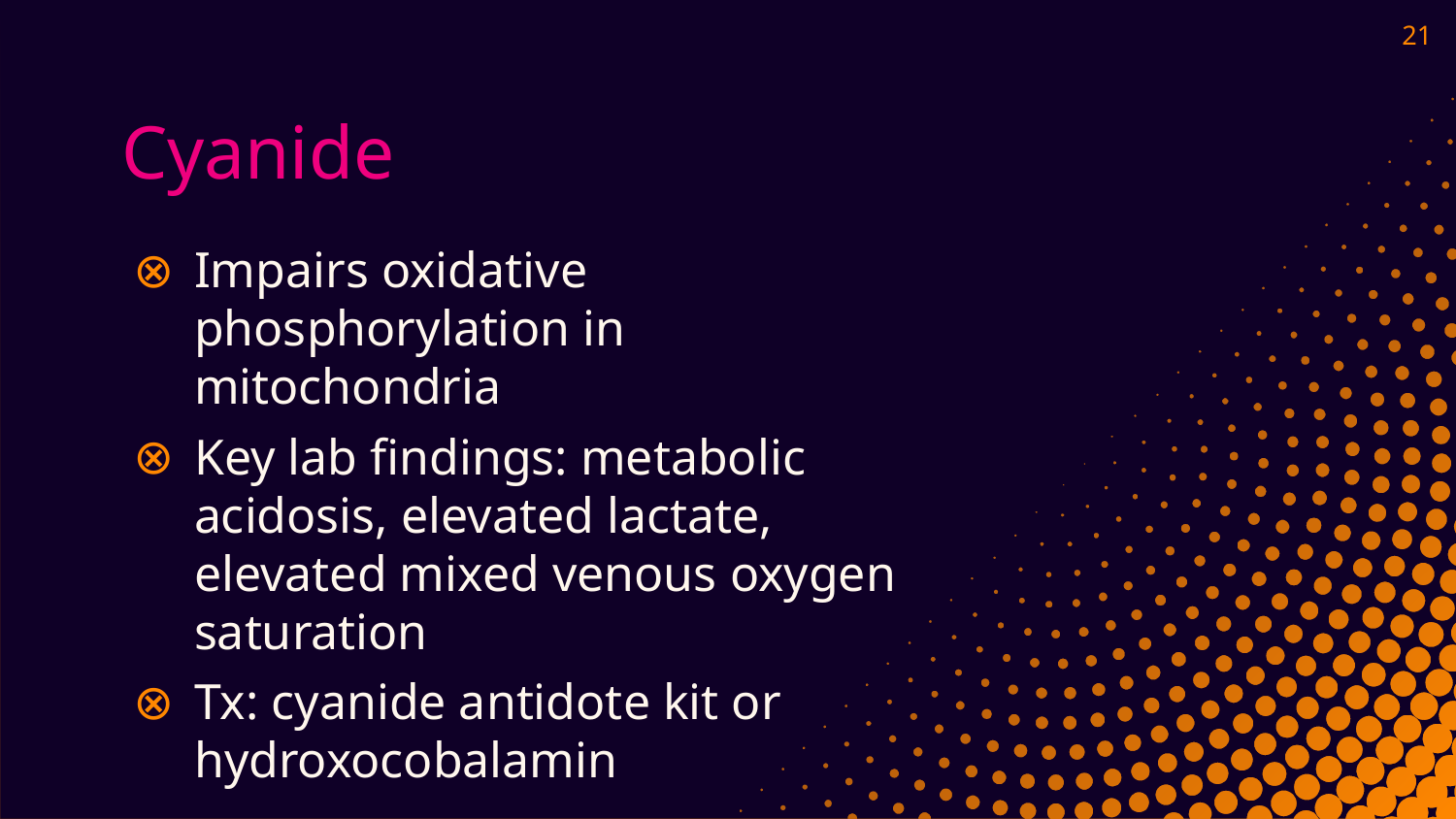

21
# Cyanide
Impairs oxidative phosphorylation in mitochondria
Key lab findings: metabolic acidosis, elevated lactate, elevated mixed venous oxygen saturation
Tx: cyanide antidote kit or hydroxocobalamin

## Slide 22
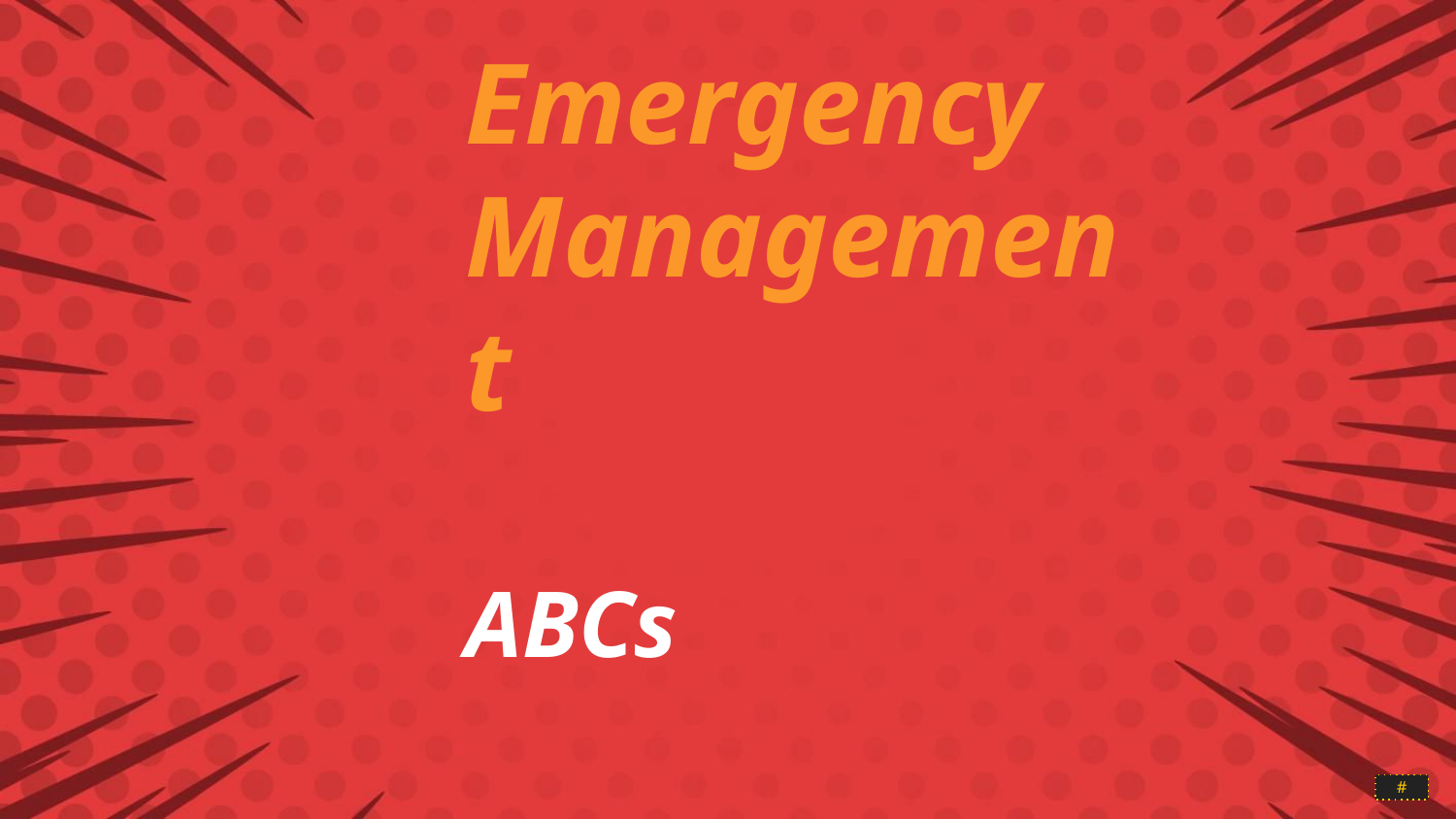

Emergency Management
ABCs
#

## Slide 23
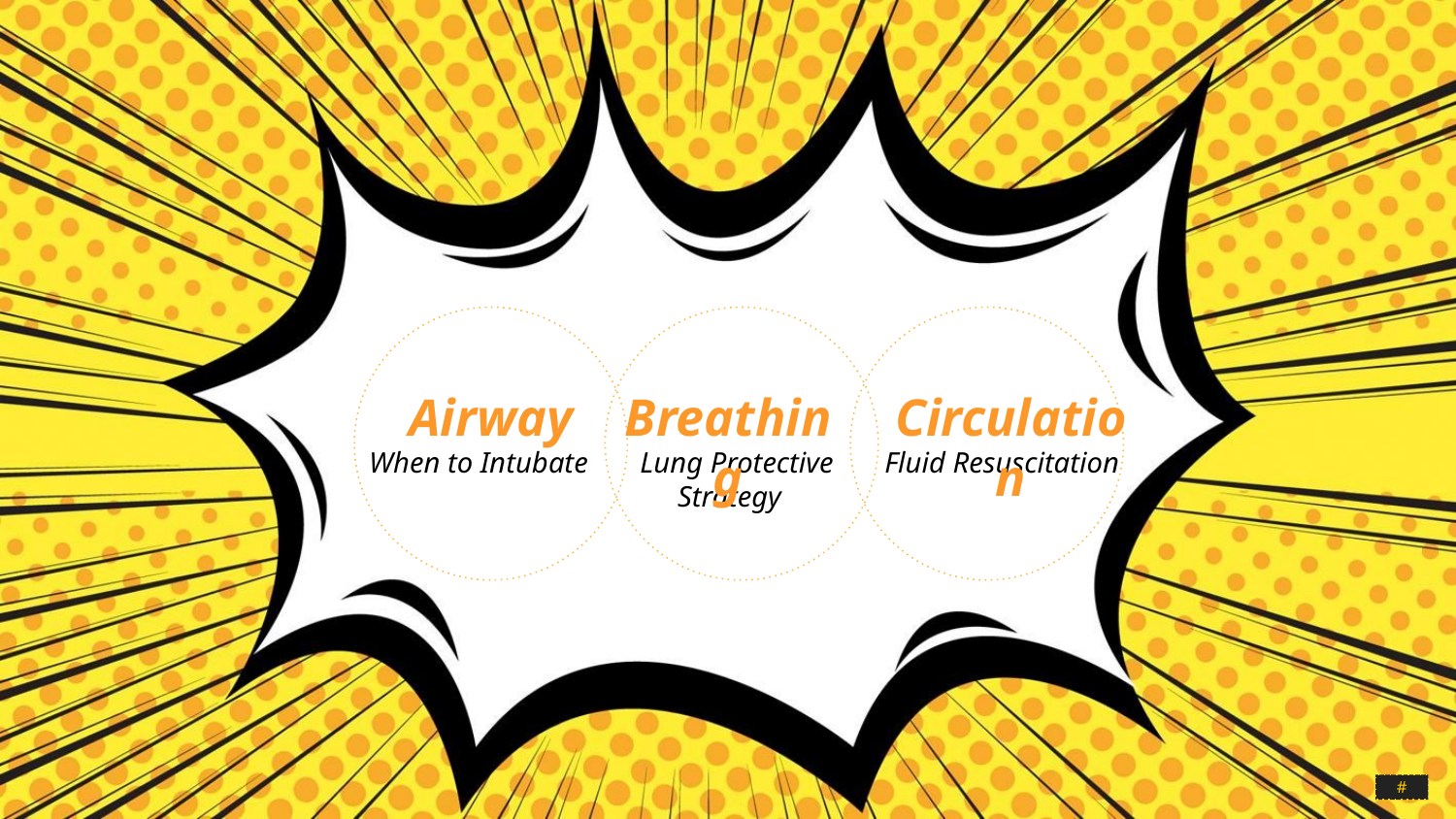

Airway
Breathing
Circulation
When to Intubate
 Lung Protective Strategy
Fluid Resuscitation
#

## Slide 24
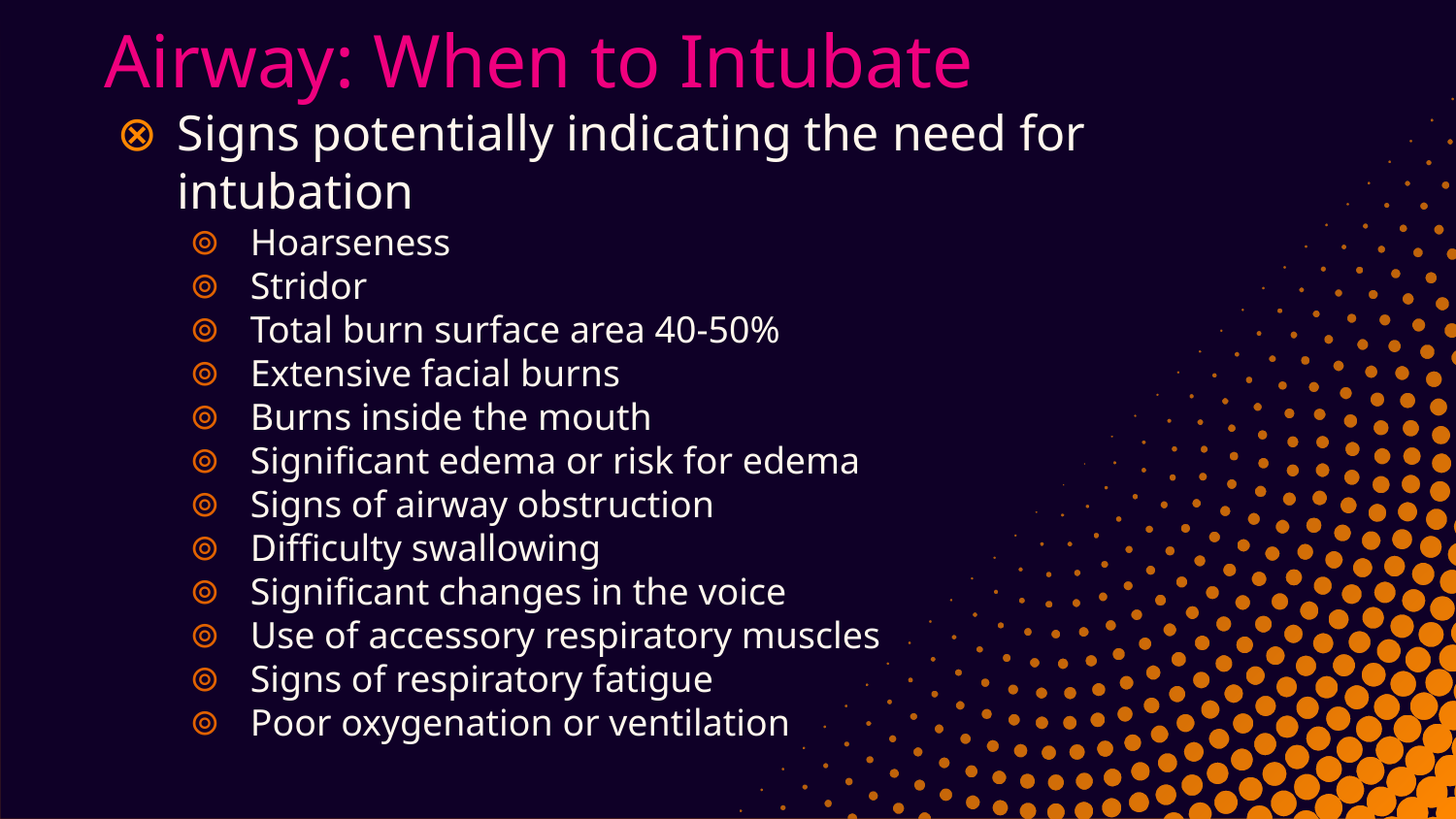

# Airway: When to Intubate
Signs potentially indicating the need for intubation
Hoarseness
Stridor
Total burn surface area 40-50%
Extensive facial burns
Burns inside the mouth
Significant edema or risk for edema
Signs of airway obstruction
Difficulty swallowing
Significant changes in the voice
Use of accessory respiratory muscles
Signs of respiratory fatigue
Poor oxygenation or ventilation

## Slide 25
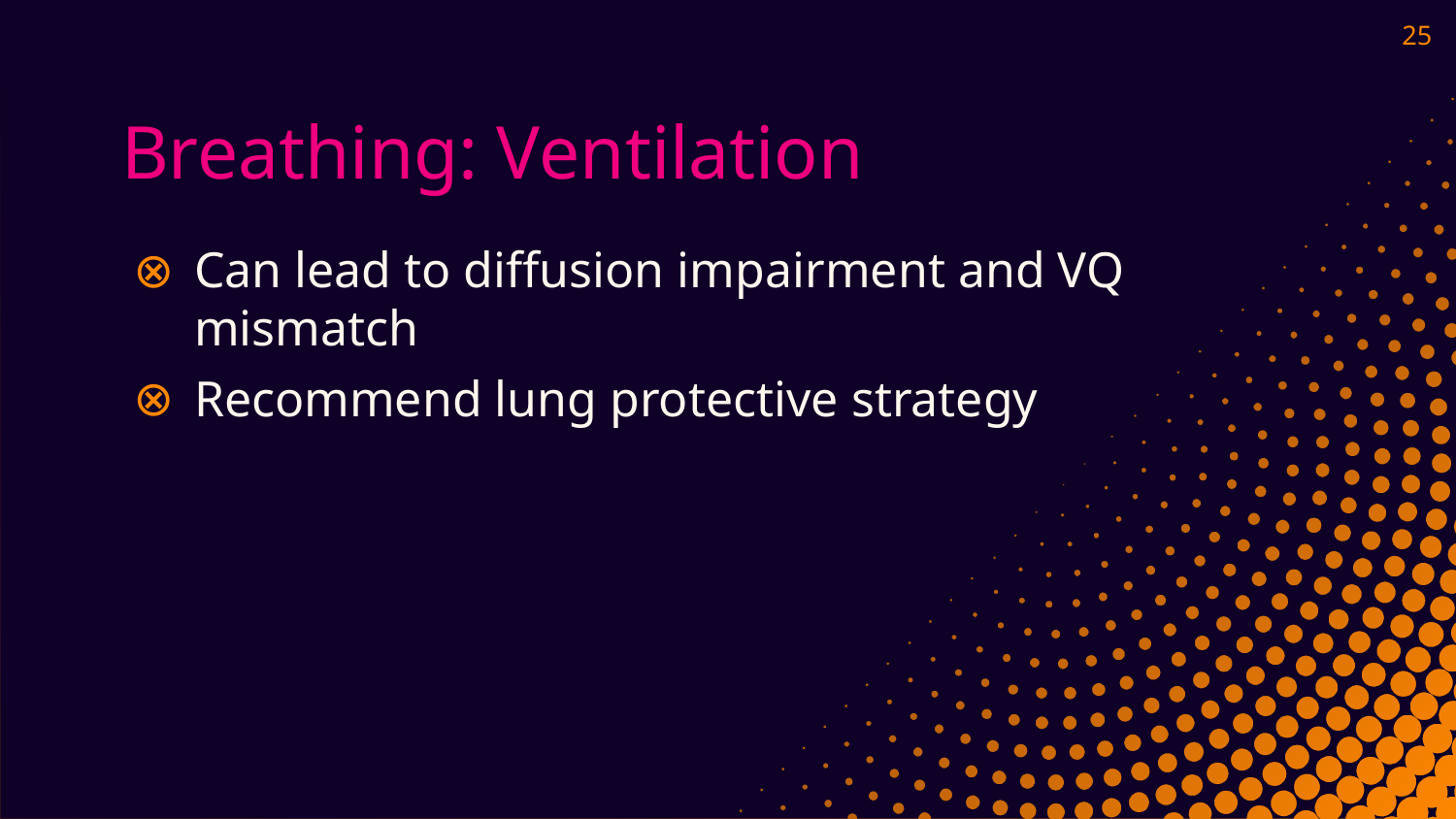

25
# Breathing: Ventilation
Can lead to diffusion impairment and VQ mismatch
Recommend lung protective strategy

## Slide 26
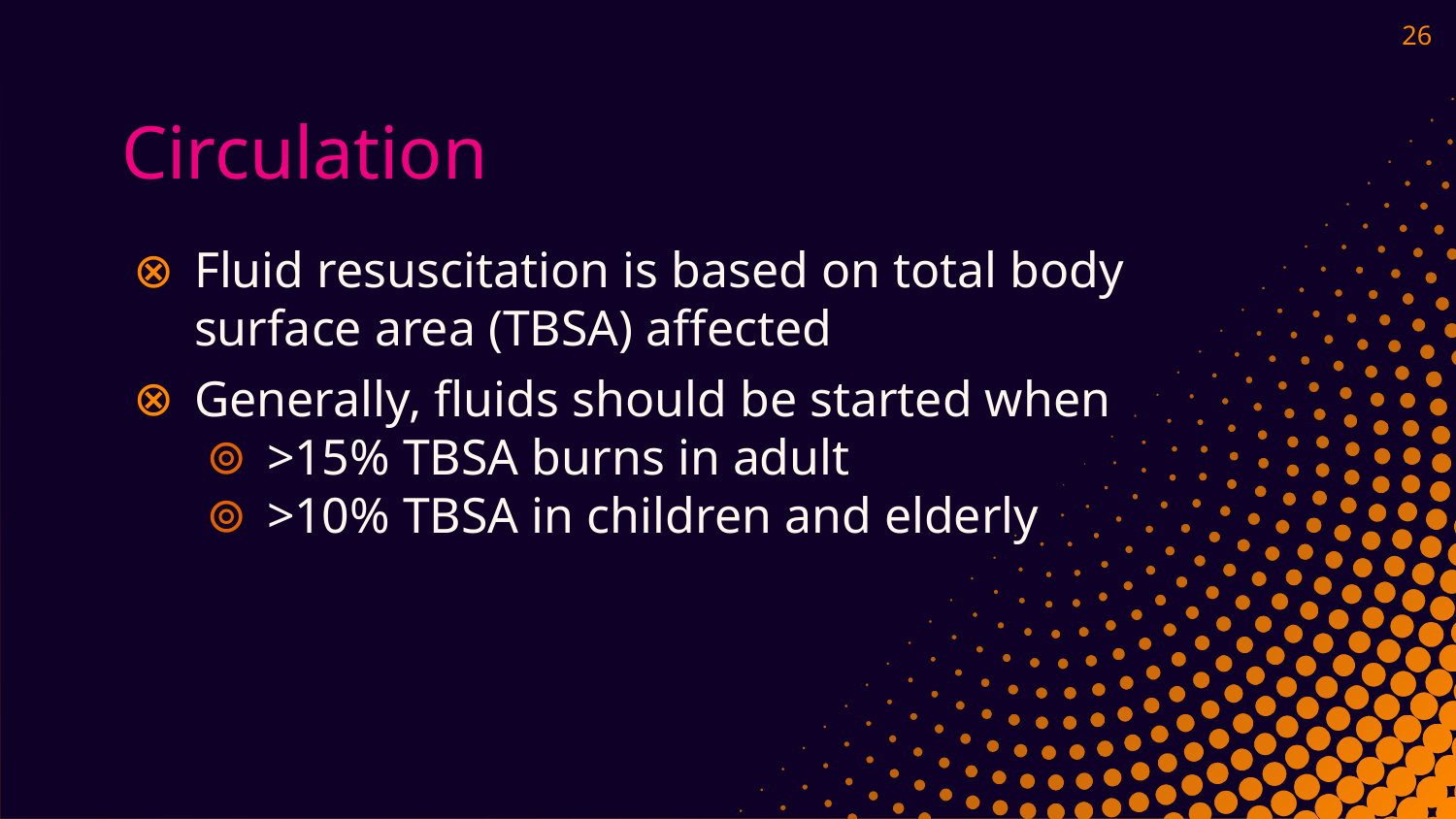

26
# Circulation
Fluid resuscitation is based on total body surface area (TBSA) affected
Generally, fluids should be started when
>15% TBSA burns in adult
>10% TBSA in children and elderly

## Slide 27
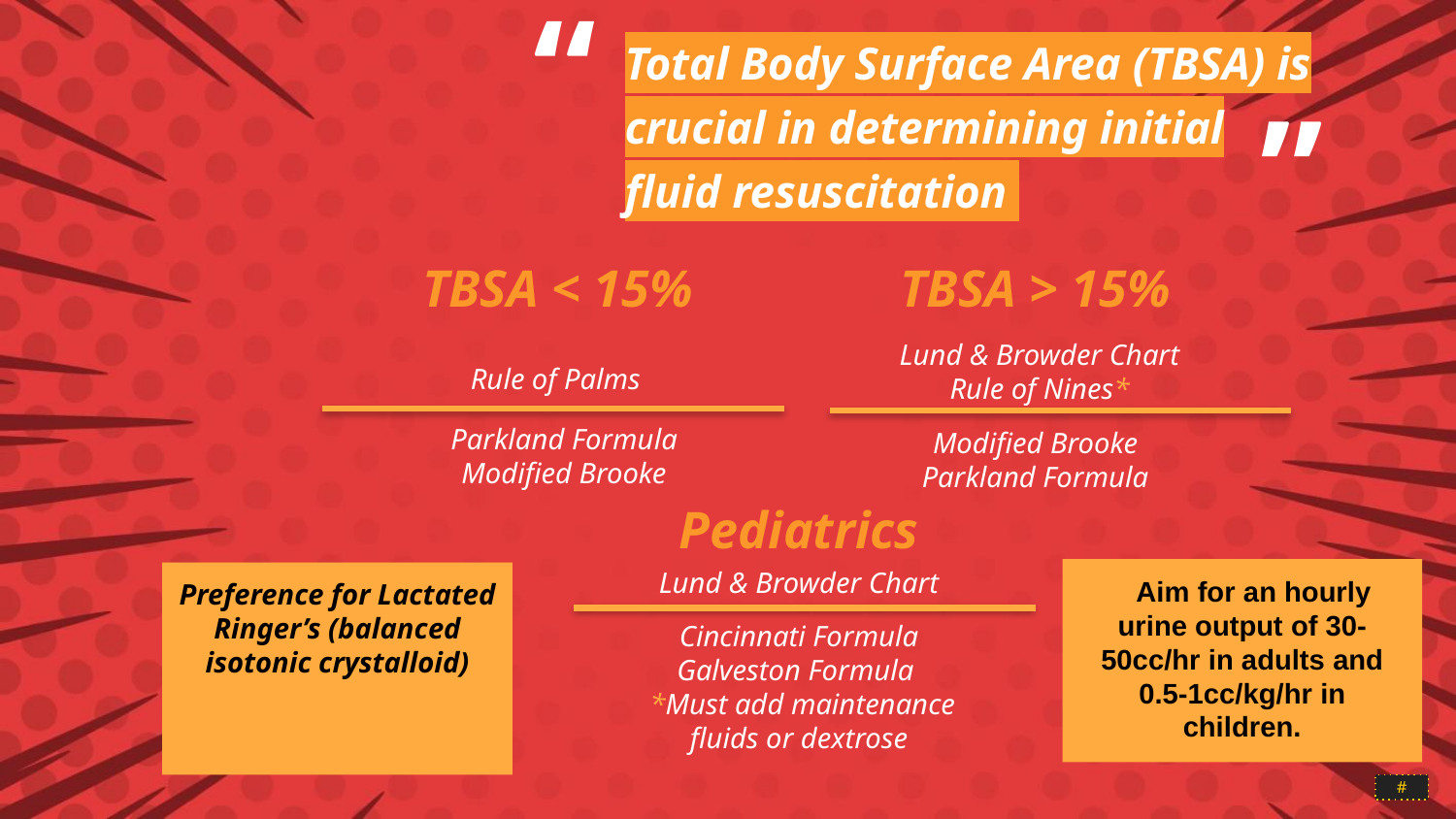

Total Body Surface Area (TBSA) is crucial in determining initial fluid resuscitation
“
“
TBSA < 15%
TBSA > 15%
Lund & Browder Chart
Rule of Nines*
Rule of Palms
Parkland Formula
Modified Brooke
Modified Brooke
Parkland Formula
Pediatrics
Lund & Browder Chart
  Aim for an hourly urine output of 30-50cc/hr in adults and 0.5-1cc/kg/hr in children.
Preference for Lactated Ringer’s (balanced isotonic crystalloid)
Cincinnati Formula
Galveston Formula
 *Must add maintenance fluids or dextrose
#

## Slide 28
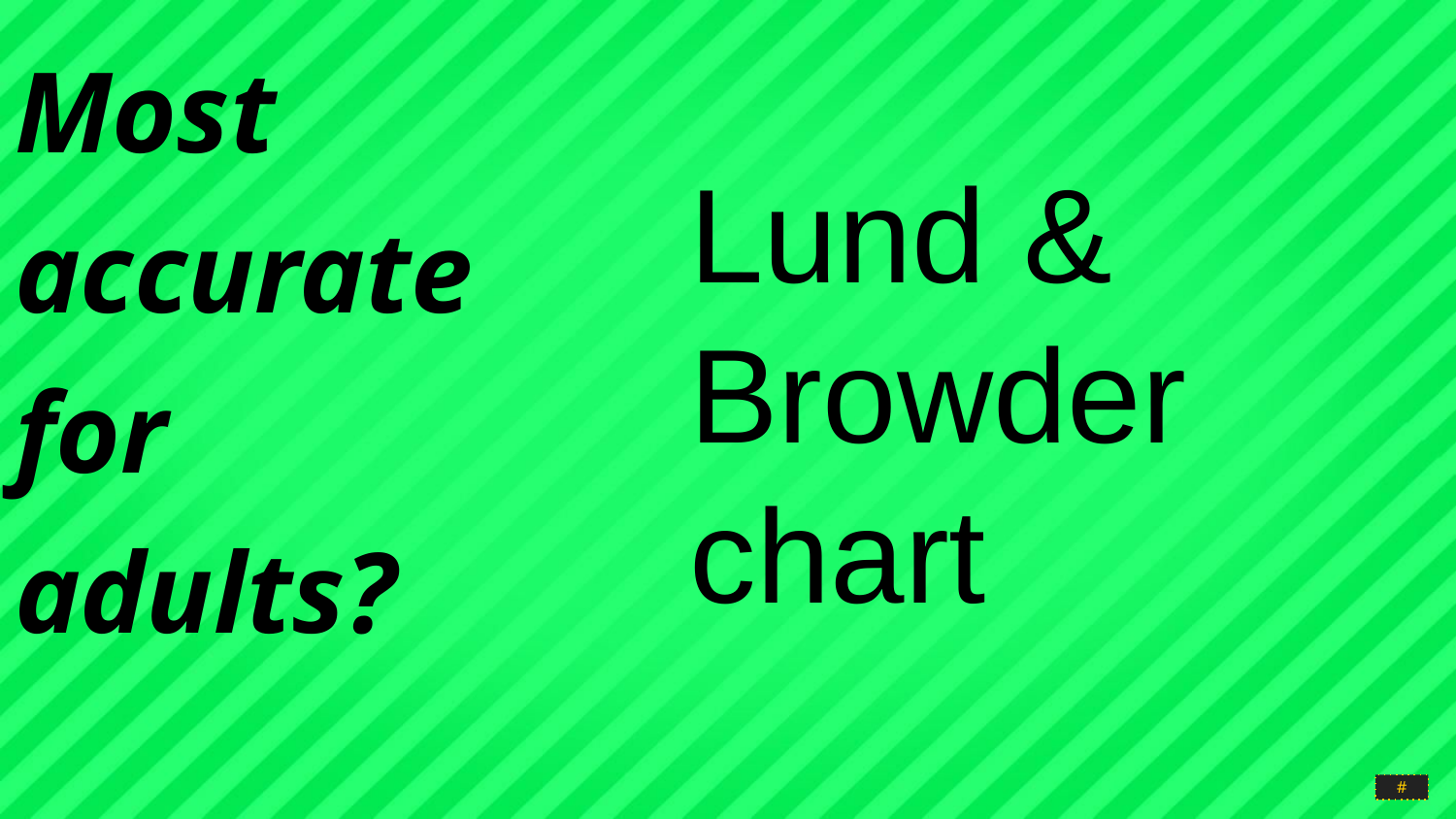

Most accurate for adults?
Lund & Browder chart
#

## Slide 29
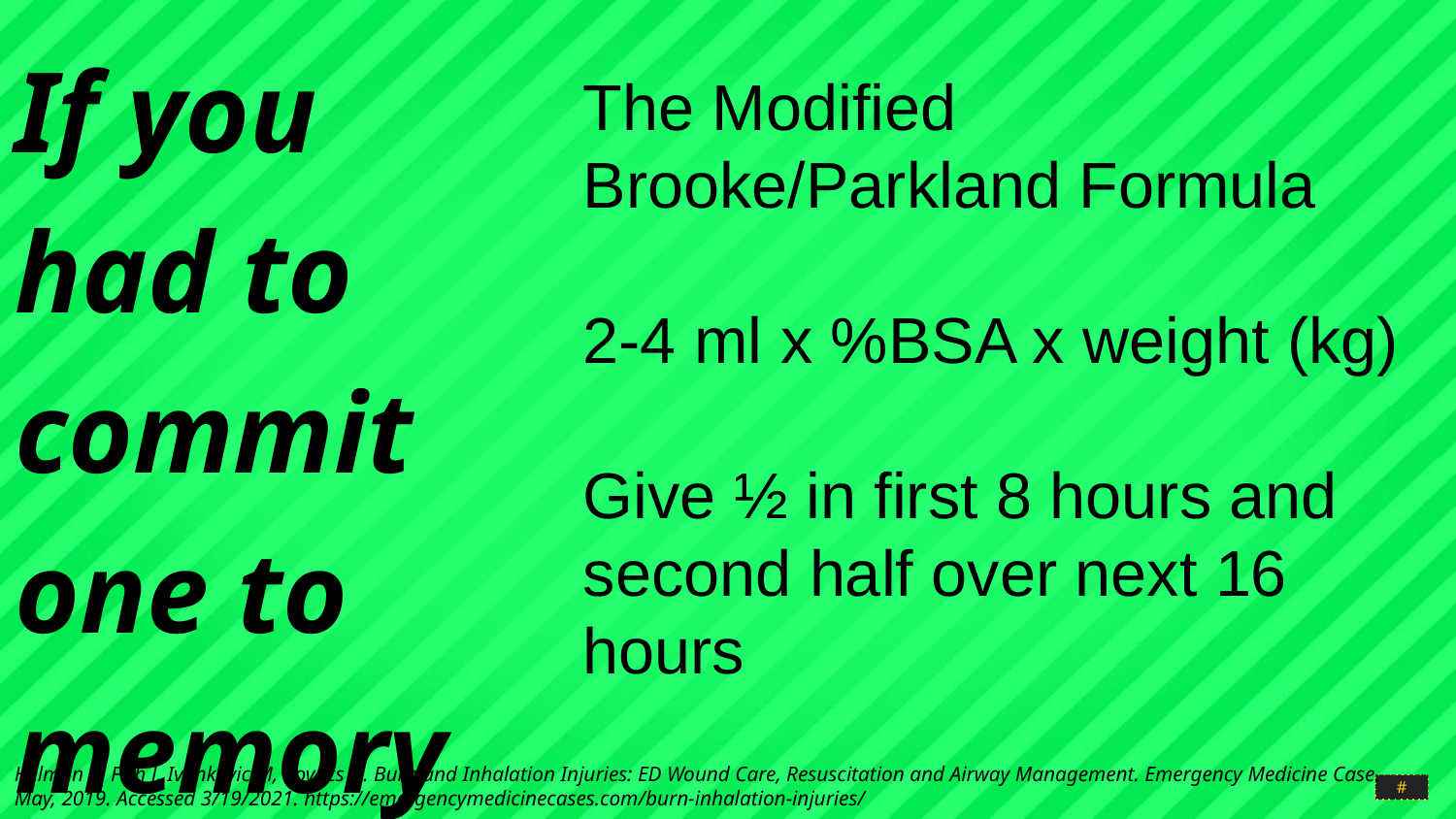

If you had to commit one to memory?
The Modified Brooke/Parkland Formula
2-4 ml x %BSA x weight (kg)
Give ½ in first 8 hours and second half over next 16 hours
Helman A, Fish J, Ivankovic M, Kovacs G. Burn and Inhalation Injuries: ED Wound Care, Resuscitation and Airway Management. Emergency Medicine Case. May, 2019. Accessed 3/19/2021. https://emergencymedicinecases.com/burn-inhalation-injuries/
#

## Slide 30
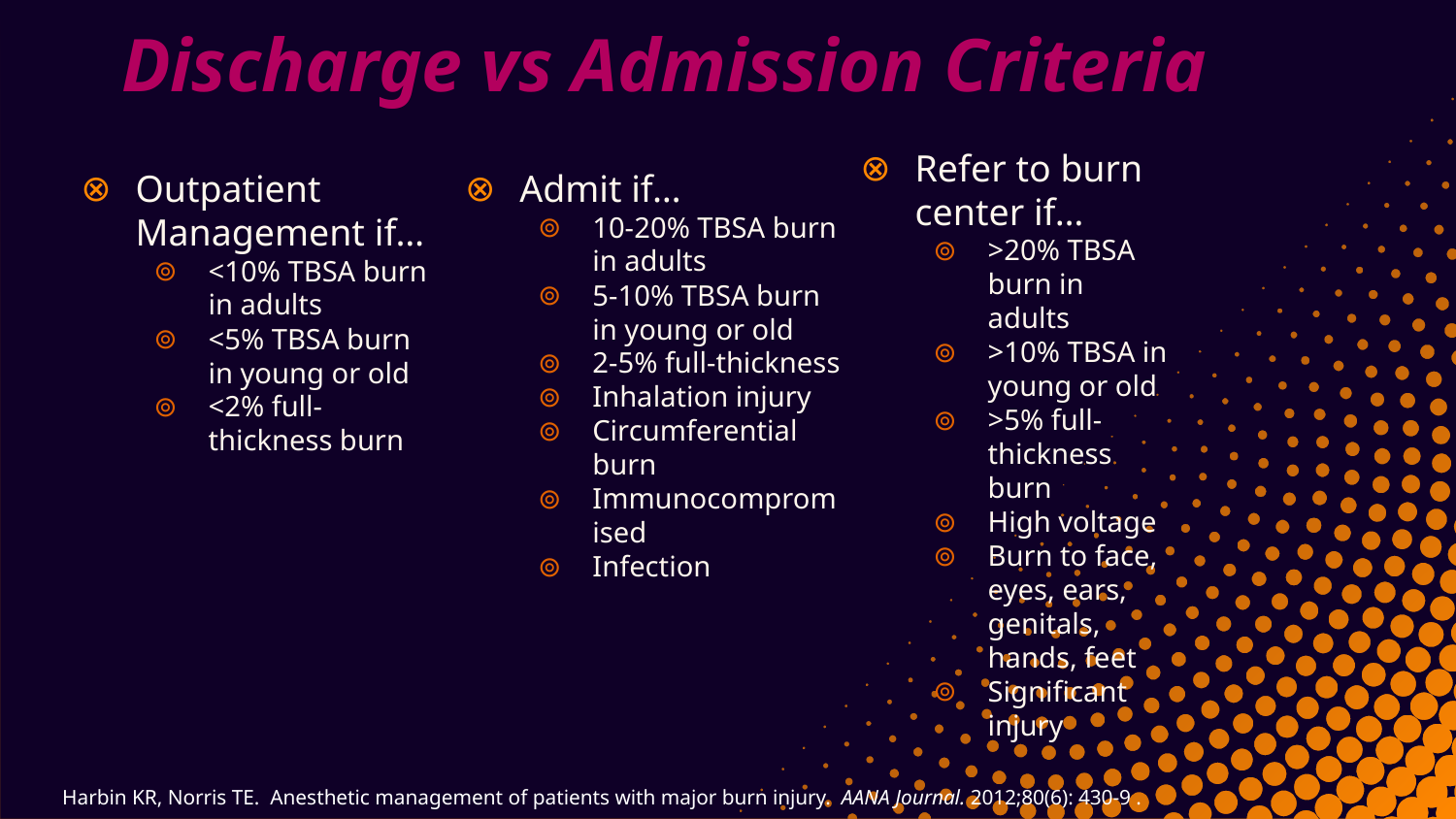

Discharge vs Admission Criteria
# Discharge vs Admission Criteria
Refer to burn center if…
>20% TBSA burn in adults
>10% TBSA in young or old
>5% full-thickness burn
High voltage
Burn to face, eyes, ears, genitals, hands, feet
Significant injury
Outpatient Management if…
<10% TBSA burn in adults
<5% TBSA burn in young or old
<2% full-thickness burn
Admit if…
10-20% TBSA burn in adults
5-10% TBSA burn in young or old
2-5% full-thickness
Inhalation injury
Circumferential burn
Immunocompromised
Infection
Harbin KR, Norris TE. Anesthetic management of patients with major burn injury.  AANA Journal. 2012;80(6): 430-9 .

## Slide 31
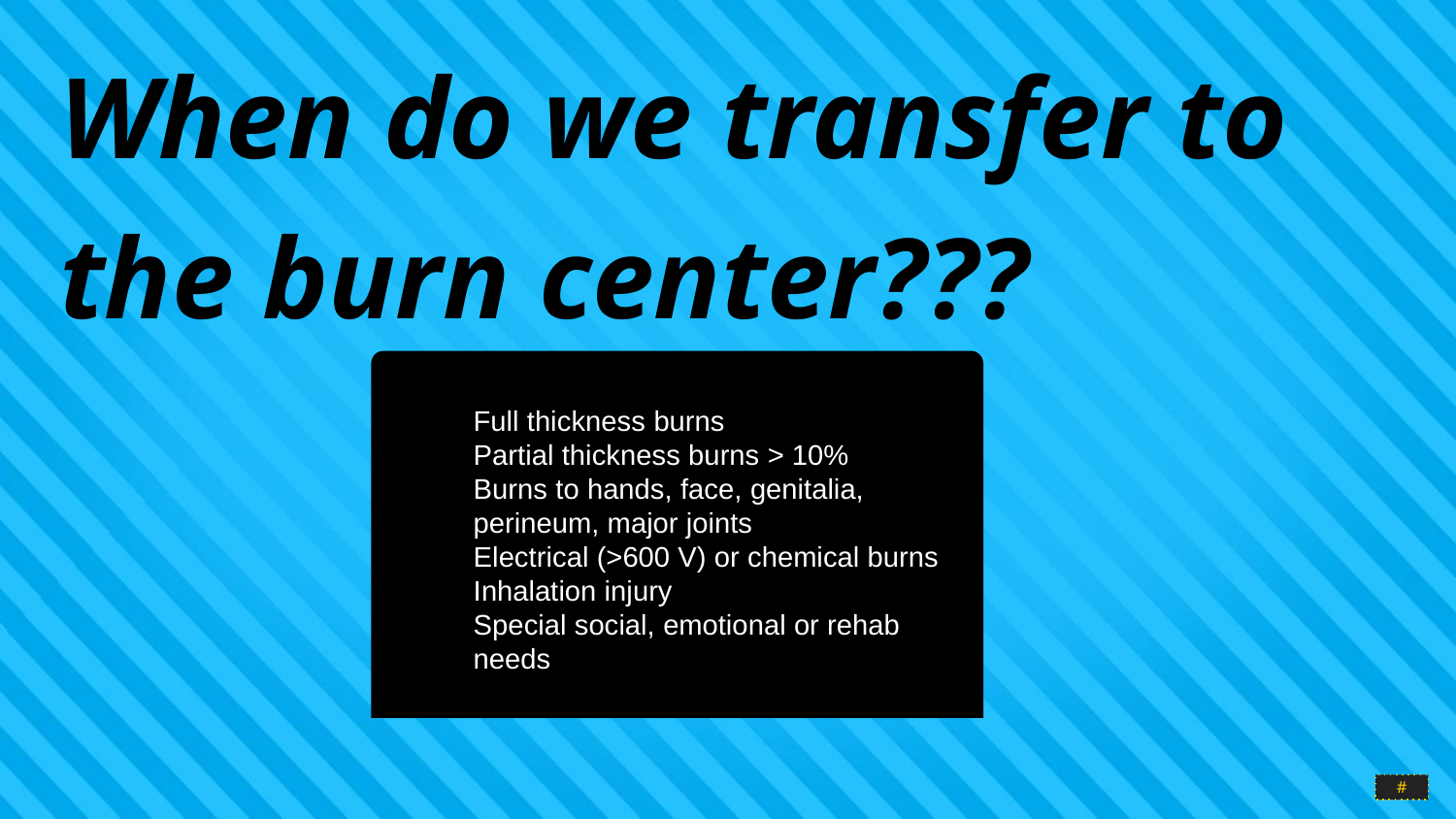

When do we transfer to the burn center???
Full thickness burns
Partial thickness burns > 10%
Burns to hands, face, genitalia, perineum, major joints
Electrical (>600 V) or chemical burns
Inhalation injury
Special social, emotional or rehab needs
#

## Slide 32
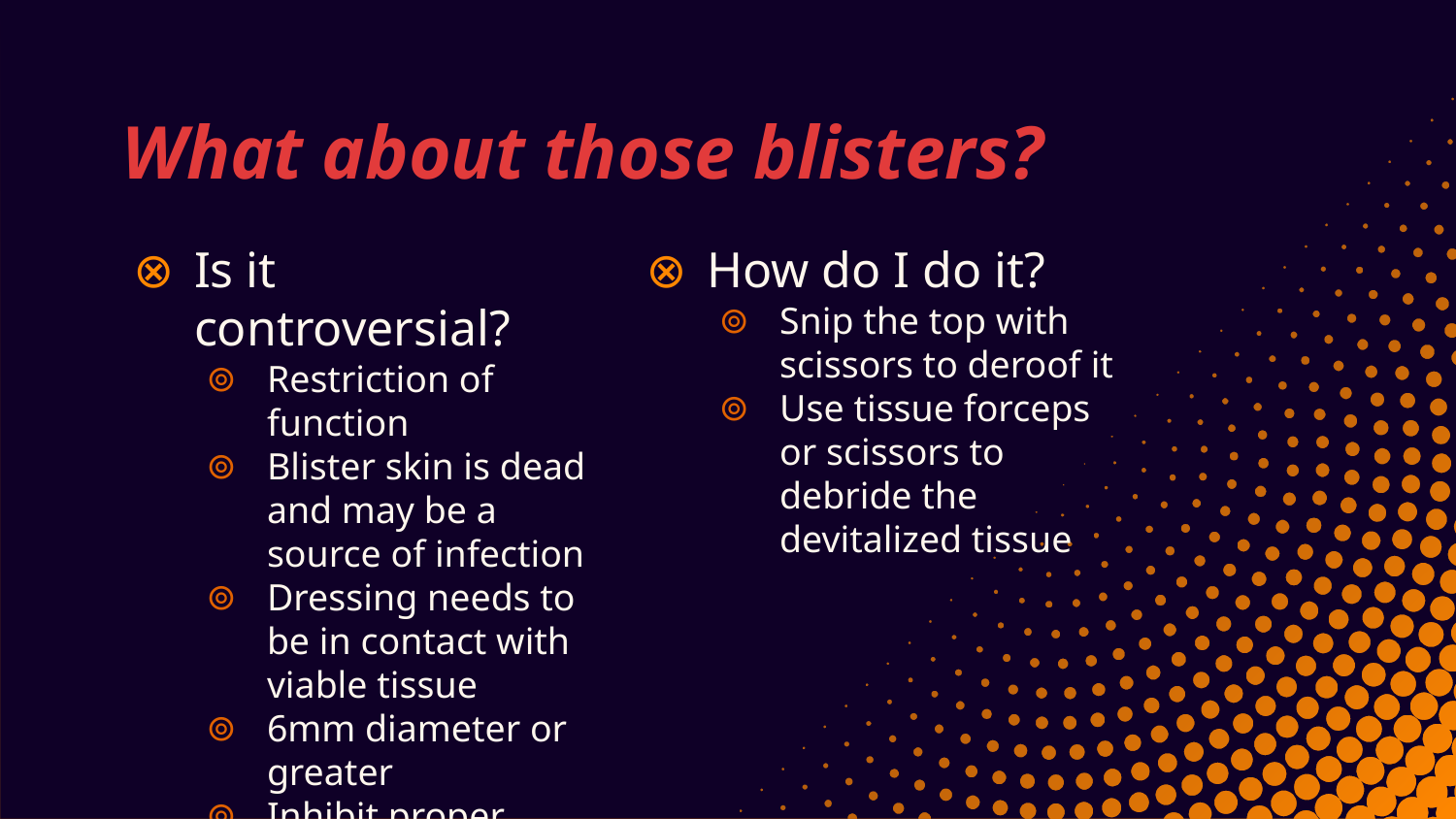

# What about those blisters?
Is it controversial?
Restriction of function
Blister skin is dead and may be a source of infection
Dressing needs to be in contact with viable tissue
6mm diameter or greater
Inhibit proper assessment.
How do I do it?
Snip the top with scissors to deroof it
Use tissue forceps or scissors to debride the devitalized tissue

## Slide 33
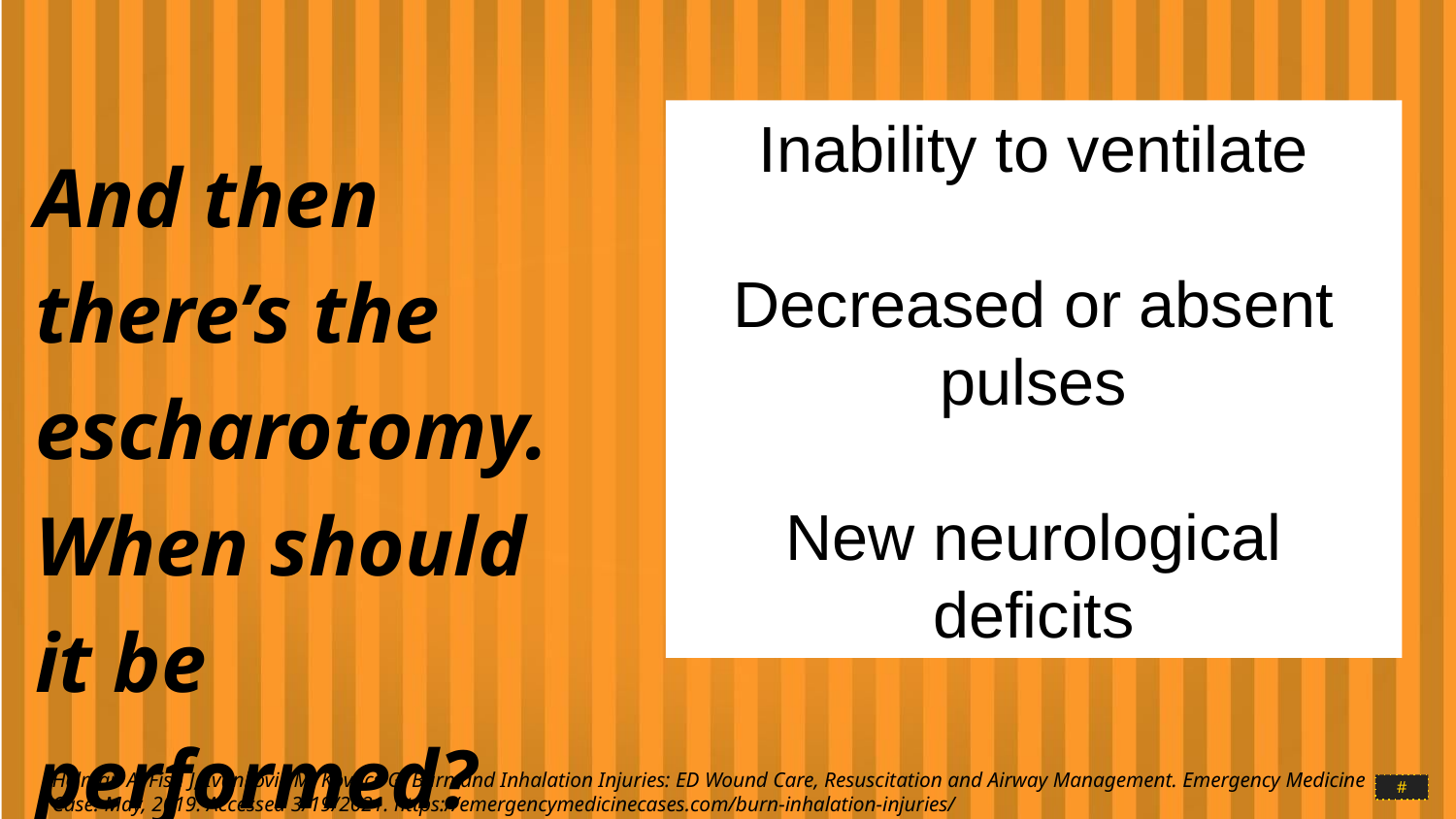

Inability to ventilate
Decreased or absent pulses
New neurological deficits
And then there’s the escharotomy. When should it be performed?
Helman A, Fish J, Ivankovic M, Kovacs G. Burn and Inhalation Injuries: ED Wound Care, Resuscitation and Airway Management. Emergency Medicine Case. May, 2019. Accessed 3/19/2021. https://emergencymedicinecases.com/burn-inhalation-injuries/
#

## Slide 34
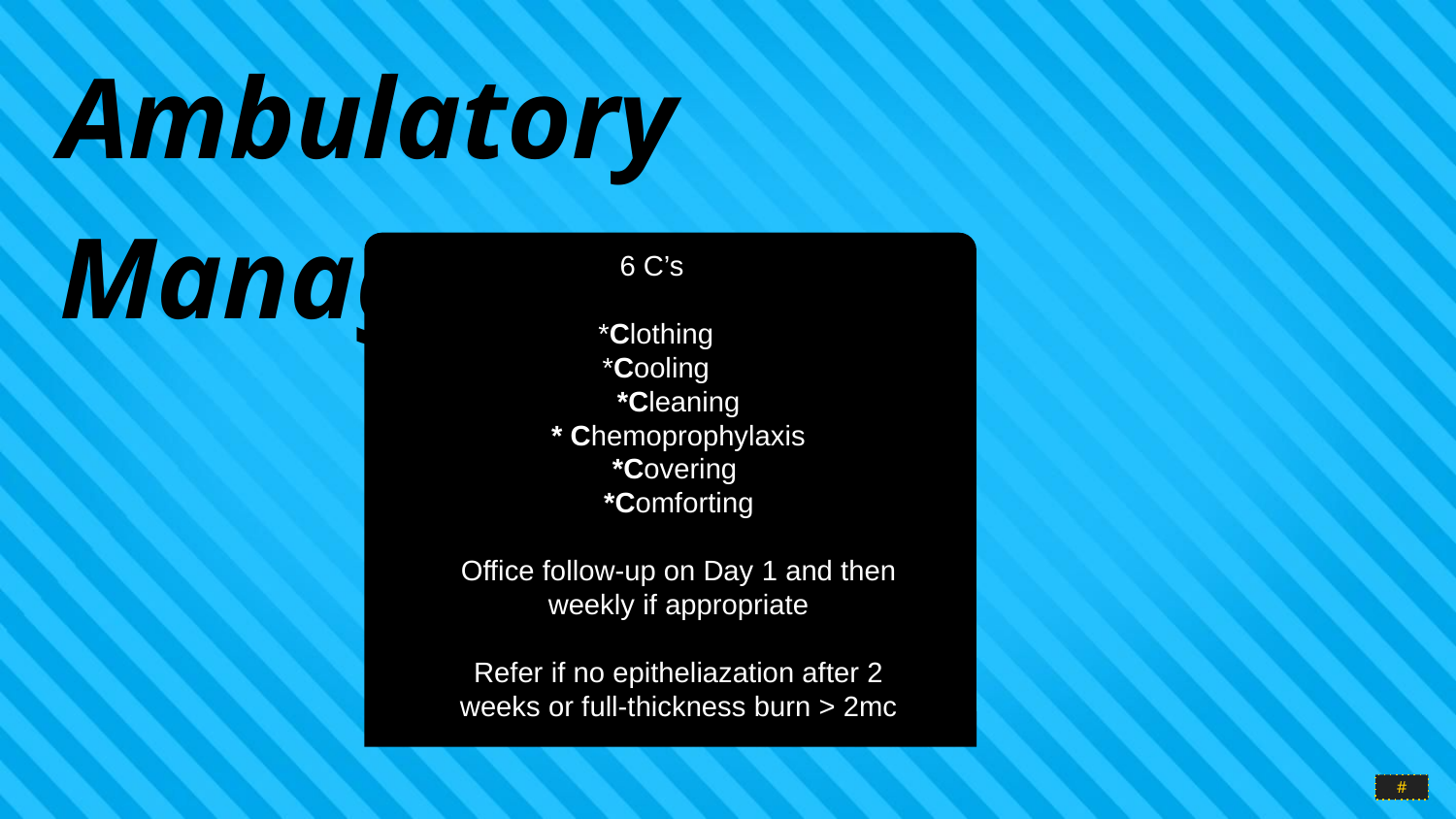

Ambulatory Management
6 C’s
*Clothing
*Cooling
*Cleaning
* Chemoprophylaxis
*Covering
*Comforting
Office follow-up on Day 1 and then weekly if appropriate
Refer if no epitheliazation after 2 weeks or full-thickness burn > 2mc
#

## Slide 35
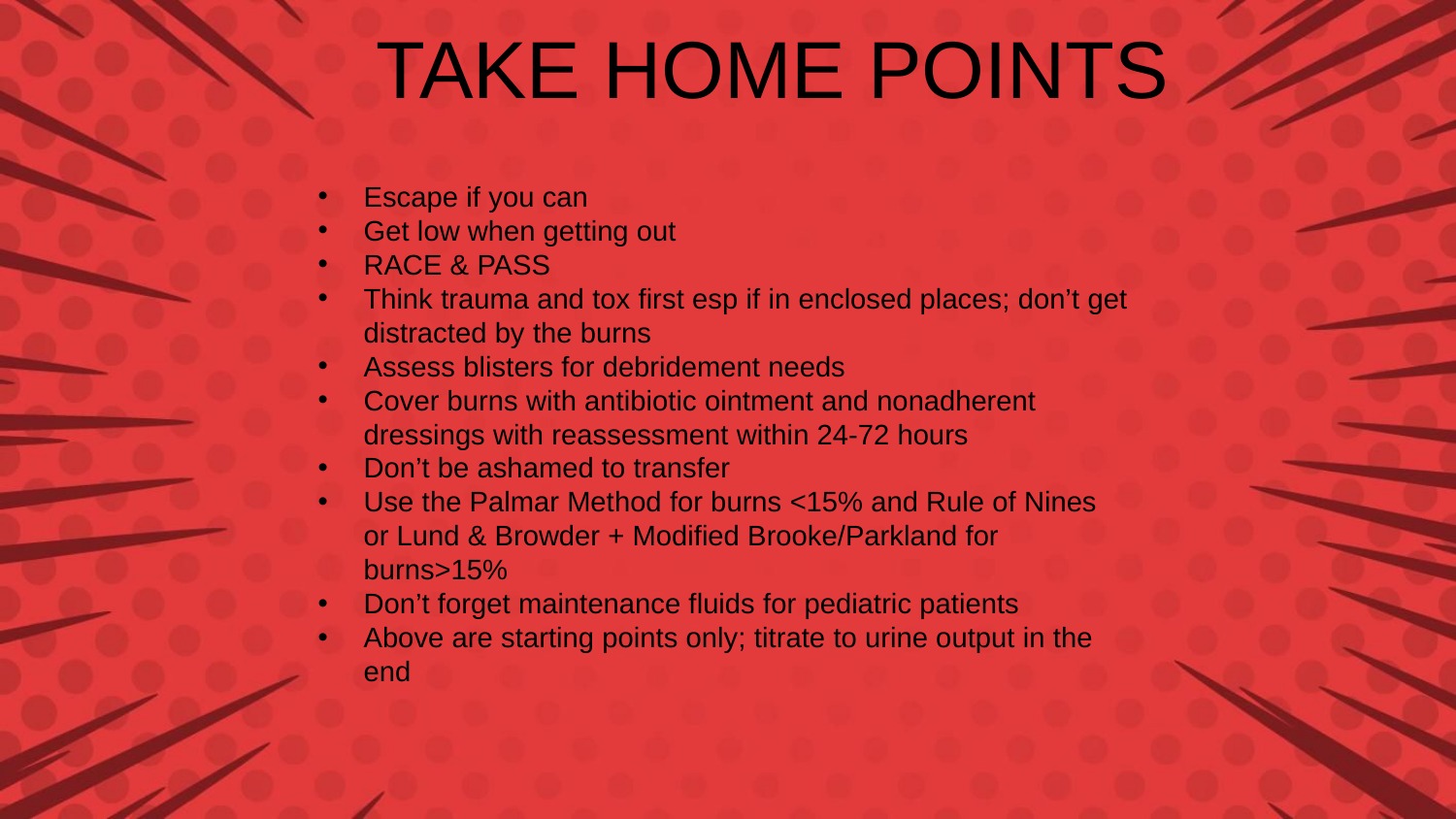

TAKE HOME POINTS
Escape if you can
Get low when getting out
RACE & PASS
Think trauma and tox first esp if in enclosed places; don’t get distracted by the burns
Assess blisters for debridement needs
Cover burns with antibiotic ointment and nonadherent dressings with reassessment within 24-72 hours
Don’t be ashamed to transfer
Use the Palmar Method for burns <15% and Rule of Nines or Lund & Browder + Modified Brooke/Parkland for burns>15%
Don’t forget maintenance fluids for pediatric patients
Above are starting points only; titrate to urine output in the end

## Slide 36
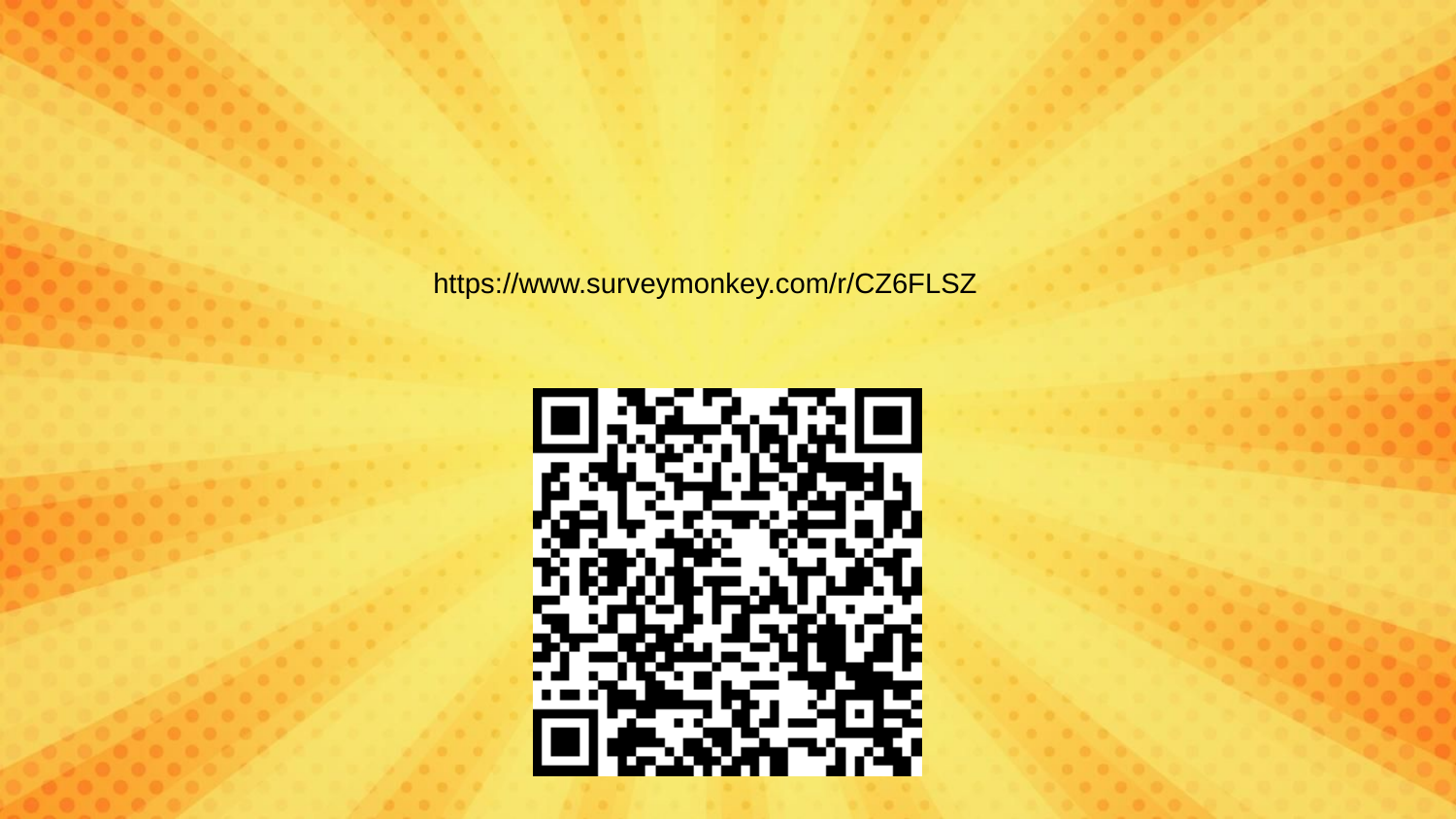

https://www.surveymonkey.com/r/CZ6FLSZ

## Slide 37
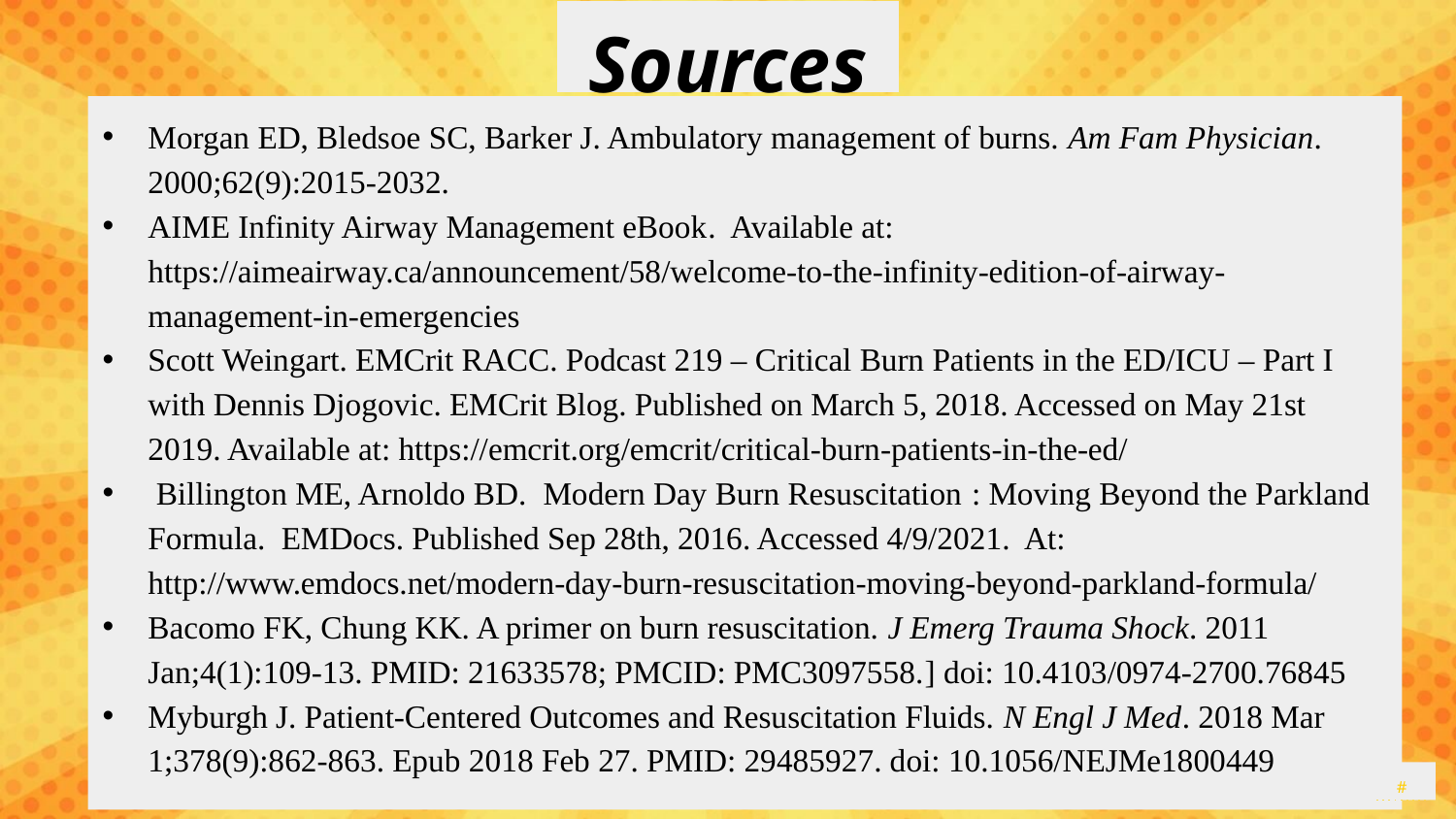

Sources
Morgan ED, Bledsoe SC, Barker J. Ambulatory management of burns. Am Fam Physician. 2000;62(9):2015‐2032.
AIME Infinity Airway Management eBook. Available at: https://aimeairway.ca/announcement/58/welcome-to-the-infinity-edition-of-airway-management-in-emergencies
Scott Weingart. EMCrit RACC. Podcast 219 – Critical Burn Patients in the ED/ICU – Part I with Dennis Djogovic. EMCrit Blog. Published on March 5, 2018. Accessed on May 21st 2019. Available at: https://emcrit.org/emcrit/critical-burn-patients-in-the-ed/
 Billington ME, Arnoldo BD. Modern Day Burn Resuscitation : Moving Beyond the Parkland Formula. EMDocs. Published Sep 28th, 2016. Accessed 4/9/2021. At: http://www.emdocs.net/modern-day-burn-resuscitation-moving-beyond-parkland-formula/
Bacomo FK, Chung KK. A primer on burn resuscitation. J Emerg Trauma Shock. 2011 Jan;4(1):109-13. PMID: 21633578; PMCID: PMC3097558.] doi: 10.4103/0974-2700.76845
Myburgh J. Patient-Centered Outcomes and Resuscitation Fluids. N Engl J Med. 2018 Mar 1;378(9):862-863. Epub 2018 Feb 27. PMID: 29485927. doi: 10.1056/NEJMe1800449
#

## Slide 38
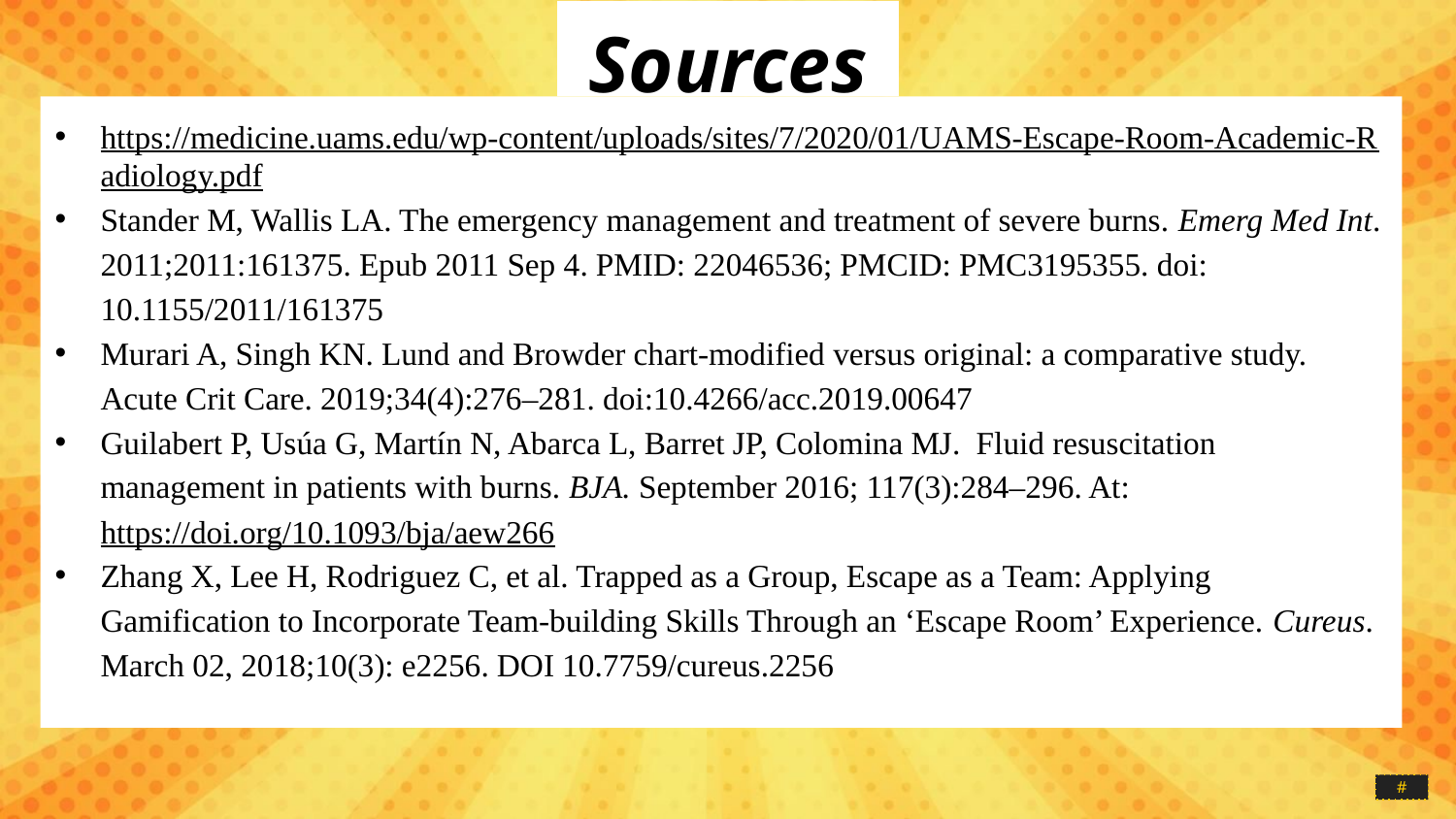

Sources
https://medicine.uams.edu/wp-content/uploads/sites/7/2020/01/UAMS-Escape-Room-Academic-Radiology.pdf
Stander M, Wallis LA. The emergency management and treatment of severe burns. Emerg Med Int. 2011;2011:161375. Epub 2011 Sep 4. PMID: 22046536; PMCID: PMC3195355. doi: 10.1155/2011/161375
Murari A, Singh KN. Lund and Browder chart-modified versus original: a comparative study. Acute Crit Care. 2019;34(4):276–281. doi:10.4266/acc.2019.00647
Guilabert P, Usúa G, Martín N, Abarca L, Barret JP, Colomina MJ. Fluid resuscitation management in patients with burns. BJA. September 2016; 117(3):284–296. At: https://doi.org/10.1093/bja/aew266
Zhang X, Lee H, Rodriguez C, et al. Trapped as a Group, Escape as a Team: Applying Gamification to Incorporate Team-building Skills Through an ‘Escape Room’ Experience. Cureus. March 02, 2018;10(3): e2256. DOI 10.7759/cureus.2256
#

## Slide 39
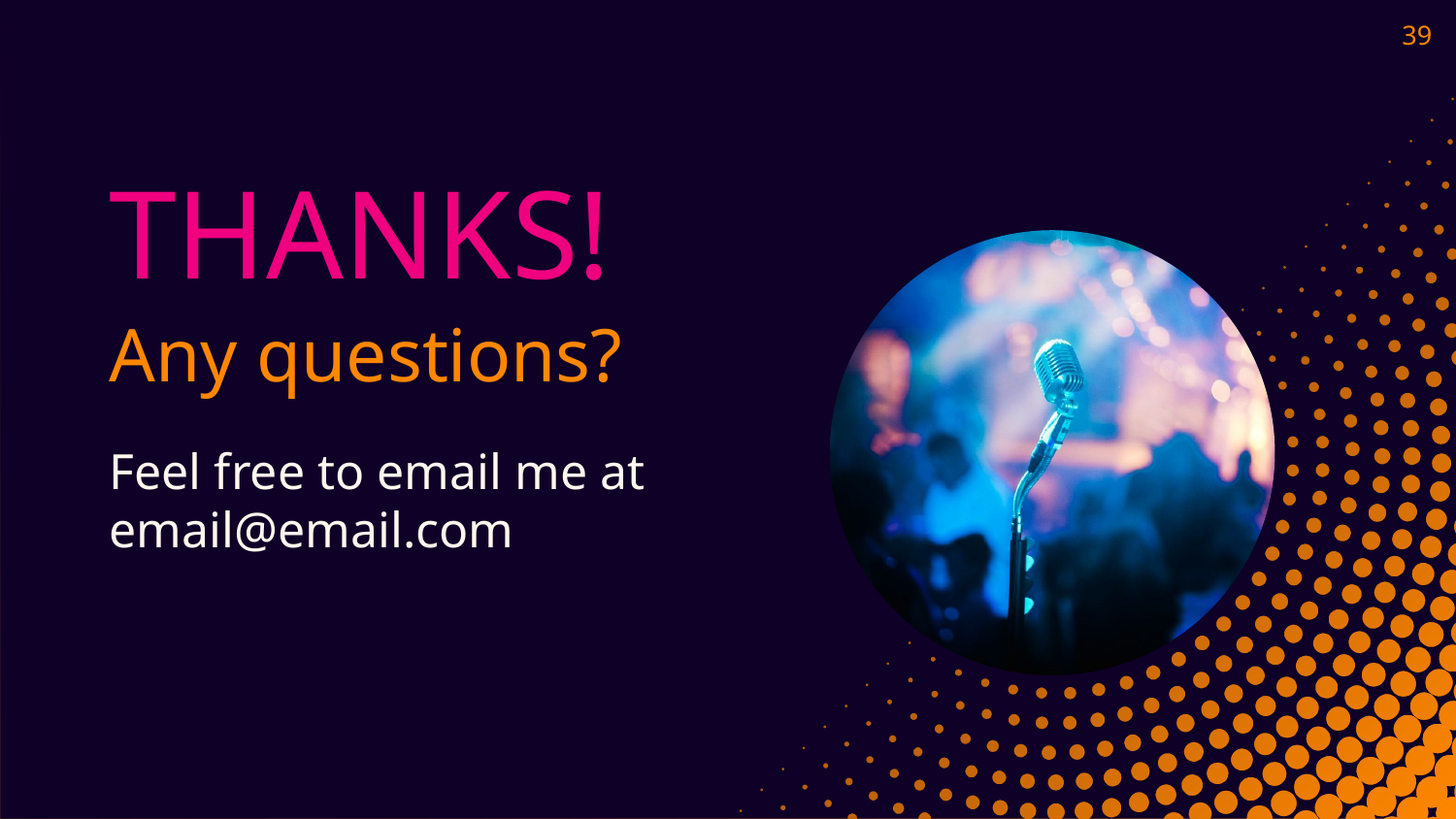

39
THANKS!
Any questions?
Feel free to email me at email@email.com
